# Metabolic State Determines Sensitivity to Cellular Stress in Huntington Disease: Normalization by Activation of PPARγ

**DOI:** 10.1371/journal.pone.0030406

**Published:** 2012-01-20

**Authors:** Youngnam N. Jin, Woong Y. Hwang, Chulman Jo, Gail V. W. Johnson

**Affiliations:** 1 Department of Pharmacology and Physiology, University of Rochester, Rochester, New York, United States of America; 2 Department of Anesthesiology, University of Rochester, Rochester, New York, United States of America; University of Medicine and Dentistry of New Jersey, United States of America

## Abstract

Impairments in mitochondria and transcription are important factors in the pathogenesis of Huntington disease (HD), a neurodegenerative disease caused by a polyglutamine expansion in the huntingtin protein. This study investigated the effect of different metabolic states and peroxisome proliferator-activated receptor γ (PPARγ) activation on sensitivity to cellular stressors such as H_2_O_2_ or thapsigargin in HD. Striatal precursor cells expressing wild type (STHdh^Q7^) or mutant huntingtin (STHdh^Q111^) were prepared in different metabolic conditions (glucose vs. pyruvate). Due to the fact that STHdh^Q111^ cells exhibit mitochondrial deficits, we expected that in the pyruvate condition, where ATP is generated primarily by the mitochondria, there would be greater differences in cell death between the two cell types compared to the glucose condition. Intriguingly, it was the glucose condition that gave rise to greater differences in cell death. In the glucose condition, thapsigargin treatment resulted in a more rapid loss of mitochondrial membrane potential (ΔΨm), a greater activation of caspases (3, 8, and 9), and a significant increase in superoxide/reactive oxygen species (ROS) in STHdh^Q111^ compared to STHdh^Q7^, while both cell types showed similar kinetics of ΔΨm-loss and similar levels of superoxide/ROS in the pyruvate condition. This suggests that bioenergetic deficiencies are not the primary contributor to the enhanced sensitivity of STHdh^Q111^ cells to stressors compared to the STHdh^Q7^ cells. PPARγ activation significantly attenuated thapsigargin-induced cell death, concomitant with an inhibition of caspase activation, a delay in ΔΨm loss, and a reduction of superoxide/ROS generation in STHdh^Q111^ cells. Expression of mutant huntingtin in primary neurons induced superoxide/ROS, an effect that was significantly reduced by constitutively active PPARγ. These results provide significant insight into the bioenergetic disturbances in HD with PPARγ being a potential therapeutic target for HD.

## Introduction

Huntington disease (HD) is an inherited neurodegenerative disease caused by an abnormal expansion of polyglutamine in the huntingtin (Htt) protein. Neuronal degeneration in HD patients begins in the striatum, especially GABAergic medium size spiny neurons, followed by involvement of the cerebral cortex as the disease progresses [1]. Despite the discovery of the unique causative genetic mutation of Htt almost two decades ago [2] there is still no satisfactorily effective treatment, and the underlying pathogenic mechanisms of HD are still elusive. Bioenergetic deficits manifested as weight loss, muscle wasting, reduced glucose uptake in cortex and striatum, and increased incidence of diabetes have been implicated in the pathogenic progression of HD [3], [4], [5]. Importantly, an increasing number of studies have shown that mutant Htt (mHtt) results in mitochondrial impairment such as deficits in the electron transport chain, Ca^2+^ handling defects, and increased sensitivity of mitochondria to permeability transition pore (mPTP) opening [4], . Furthermore, numerous studies have demonstrated that oxidative stress plays a pivotal role in the pathogenesis of HD [8], [9], [10].

Transcriptional dysregulation has been considered a crucial pathogenic mechanism in HD [5], [11]. Many studies have reported that the nuclear localization of mHtt leads to dysregulation of transcriptional factors/cofactors including peroxisome proliferator-activated receptor γ (PPARγ) coactivator-1α (PGC-1α) [12]. PGC-1α is a master regulator of mitochondrial functions as it regulates the expression of genes involved in mitochondrial bioenergetics and respiration, detoxification of ROS, and thermogenesis. PGC-1α is repressed in models of HD and PGC-1α expression significantly protects striatal neurons from mHtt-induced toxicity [12]. PGC-1α acts as a transcriptional coactivator via interaction with a variety of transcription factors including PPARγ of the PPAR family. PPARγ is an important regulator in adipogenesis, fatty acid oxidation, and mitochondrial function. PPARγ hetero-dimerizes with retinoid X receptor (RXR) [13]. Upon ligand binding, PPARγ transactivates the target genes with the support of coactivators including PGC-1α. Thiazolidinediones (e.g., rosiglitazone (RSG), pioglitazone, troglitazone) are exogenous PPARγ agonists which have been clinically used to treat type 2 diabetes. PPARγ activation is beneficial in the R6/2 mouse model of HD [14] as well as other models of neurological diseases [15], [16], [17], [18], [19]. Our previous study showed that PPARγ activity was severely compromised in STHdh^Q111^ cells (striatal cells expressing mHtt) [6]. Further, thapsigargin (TG) induced a loss of mitochondrial membrane potential (ΔΨm) in STHdh^Q111^ but not STHdh^Q7^ cells (striatal cells expressing Htt) and RSG treatment attenuated TG-induced ΔΨm loss in STHdh^Q111^ cells [6]. These studies suggest that transcriptional dysregulation is tightly linked with mitochondria defects and that activating the impaired transcriptional pathways is likely to have beneficial effects in HD.

Given that bioenergetic disturbance has emerged as a key component in the pathogenesis of HD, in the present study we hypothesized that different metabolic conditions (glucose vs. pyruvate) would differentially impact cell death induced by stressors such as H_2_O_2_ or TG in HD and wild type models. In addition, although we previously showed that TG-induced ΔΨm loss in STHdh^Q111^ cells was attenuated by PPARγ activation, whether PPARγ activation protects striatal cells from stress-induced cell death remained untested. Therefore, we investigated further whether the pathological changes induced by stresses can be rescued by PPARγ activation. We expected that STHdh^Q111^ cells would show greater cell death compared to STHdh^Q7^ cells, and that the pyruvate condition would exacerbate the differences in cell death between the two cell types compared to what was observed in the glucose condition because there are numerous studies showing mitochondrial impairment in HD models, including deficits in ATP production [20], [21]. Unexpectedly but intriguingly, the glucose condition resulted in much greater differences in stress-induced cell death between the two cell types. These findings suggest that the bioenergetic status of the STHdh^Q111^ cells is not a major contributor to the enhanced sensitivity to cell death stressors, and that other variables likely play a more important role. Further, PPARγ activation protected STHdh^Q111^ cells against stresses and significantly reduced superoxide/ROS generation in STHdh^Q111^ cells and primary cortical neurons expressing mHtt. These results provide important insight into the pathogenesis of HD involving transcriptional dysregulation, oxidative stress, and metabolic impairment, and suggest that PPARγ may be a potential therapeutic target for HD.

## Results

### Mutant Huntingtin Sensitizes Striatal Cells to Stressors in the Glucose Condition

**Figure 1:**
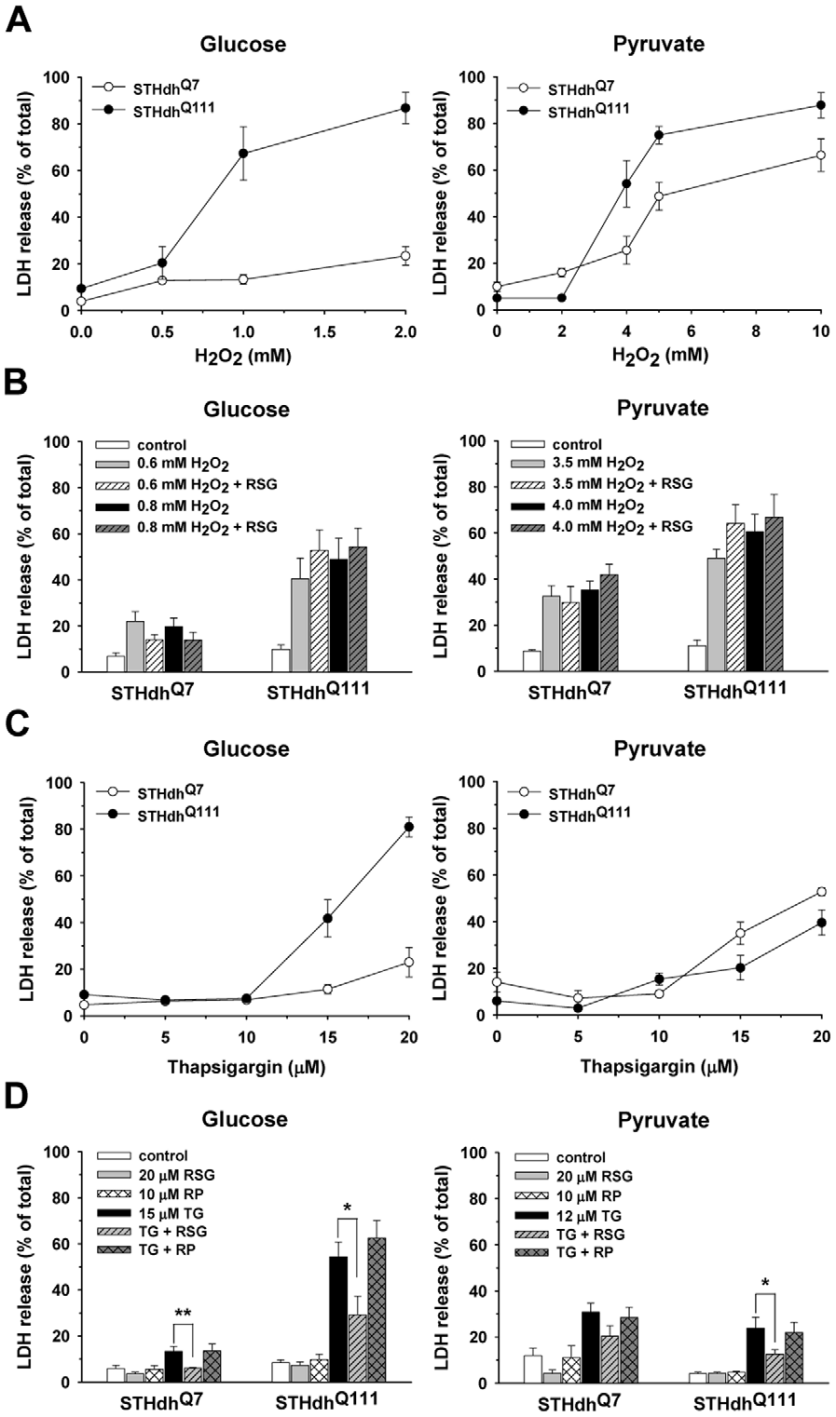
Mutant huntingtin expression sensitizes striatal cells to stressors in the glucose condition. *A*, H_2_O_2_ treatment in the glucose condition results in significantly greater cell death in STHdh^Q111^ than STHdh^Q7^ cells, while both cell types show similar cell death responses to H_2_O_2_ in pyruvate condition. *n* = 4. *B*, RSG treatment does not protect striatal cells from H_2_O_2_ toxicity. *n* = 3–4. *C*, TG treatment in the glucose condition results in much greater cell death in STHdh^Q111^ than STHdh^Q7^ cells, while both cell types show similar cell death responses to TG in the pyruvate condition. *n* = 4–6. *D*, RSG significantly attenuates TG-induced cell death in the glucose and pyruvate conditions. *n* = 3–4. RP, rolipram. Data shown are mean ± SE. * *P*<0.05, ** *P*<0.01.

**Figure 2:**
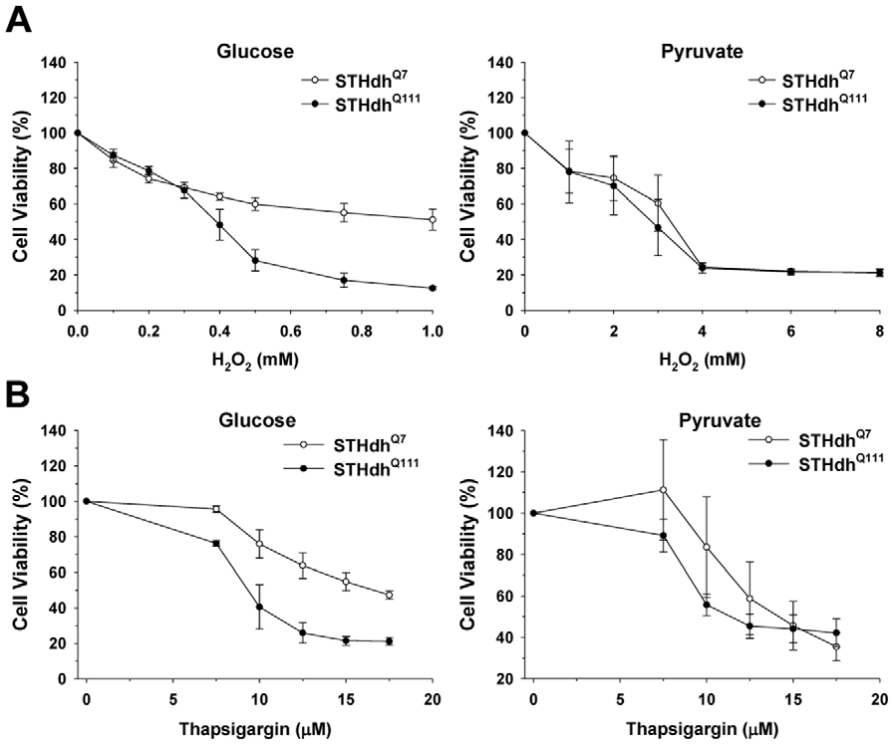
Mutant huntingtin expression exacerbates striatal cell viability loss in response to stressors in the glucose condition. Striatal cells were maintained in the different metabolic conditions. Cell viability was measured 8 h after treatment H_2_O_2_ (*A*) or TG (*B*) using the resazurin assay. Treatment with H_2_O_2_ or TG in the glucose condition significantly reduces the viability of STHdh^Q111^ cells compared to STHdh^Q7^ cells, while both cell types show similar viability responses to H_2_O_2_ or TG in the pyruvate condition. *n* = 3. Data shown are mean ± SE.

To investigate if metabolic conditions differentially affect the susceptibility of striatal cells expressing Htt or mHtt to different stressors, striatal cells were maintained in media containing glucose which supports both glycolysis and oxidative phosphorylation (Oxphos) or pyruvate which predominantly supports Oxphos [22], [23]. Striatal cells were treated with different concentrations of H_2_O_2_ or TG for 12 h and cell death was assessed by measuring LDH release. Unexpectedly, in the glucose condition STHdh^Q111^ cells showed much greater cell death in response to H_2_O_2_ or TG compared to STHdh^Q7^ cells, while in the pyruvate condition both cell types showed a similar cell death response (Fig. 1, *A* and *C*). It should be noted that LDH release may be underestimated in the pyruvate condition because pyruvate acts as a competitive inhibitor in the LDH assay [24]. Hence, it is not appropriate to compare the extent of LDH release between the glucose and pyruvate conditions. To further confirm the results from the LDH assay, cell viability was also measured using the resazurin assay (Fig. 2) [25]. As with the LDH assay (Fig. 1), greater differences in response to stressors between the two cell types were observed in the glucose condition as compared to the pyruvate condition when viability was measured with the resazurin assay (Fig. 2). Together these results demonstrate that mHtt sensitizes striatal cells to stressors in the glucose condition, while striatal cells expressing either Htt or mHtt show similar susceptibility to stressors in the pyruvate condition.

Since cells in the glucose condition utilize glycolysis as well as Oxphos, cells were treated with Oxphos inhibitors in the glucose medium to further understand how the different metabolic conditions affect the susceptibility to cellular stress (Fig. S1). STHdh^Q111^ cells in the presence of either oligomycin, a complex V inhibitor, or rotenone, a complex I inhibitor, showed greater cell death in response to H_2_O_2_ or TG compared to STHdh^Q7^ cells, suggesting that glycolysis may be a critical factor in rendering striatal cells expressing mHtt more sensitive to stressors. Since the concentration of glucose in media was high (25 mM), we also measured cell death in low (5 mM) glucose to determine if the greater difference in cell death between the two cell types in the glucose condition compared to the pyruvate condition was due to the effect of high glucose or the metabolic state. In 5 mM glucose, STHdh^Q111^ cells exhibited a similar cell death profile in response to H_2_O_2_ or TG as observed in 25 mM glucose (Fig. S2), indicating that it is the metabolic condition that contributes to the differential cell death. Next, we examined whether the PPARγ agonist, RSG attenuated cell death in response to H_2_O_2_ or TG. Striatal cells were pretreated with RSG for 24 h prior to treatment with H_2_O_2_ or TG. RSG treatment significantly reduced cell death in response to TG but not H_2_O_2_ in both glucose and pyruvate conditions (Fig. 1, *B* and *D*). We also tested whether treatment with rolipram (RP), a phosphodiesterase IV (PDE4) inhibitor, attenuated TG-induced cell death, since intracellular cAMP levels are decreased in HD models and RP treatment has beneficial effects [26], [27]. However, RP did not attenuate TG-induced cell death (Fig. 1*D*). In addition, an endogenous PPARγ agonist 15-d-PGJ_2_ also protected STHdh^Q111^ cells from TG-induced death (data not shown).

### Repressed PPARγ Activity Is Independent of the Protein Level

**Figure 3:**
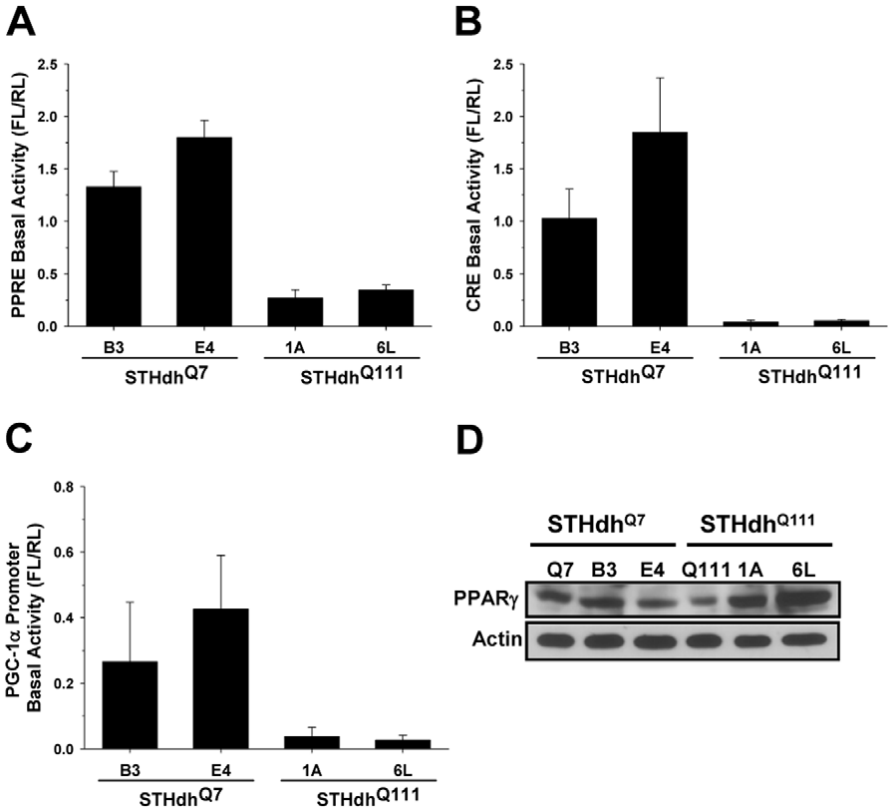
Mutant huntingtin results in repressed transcriptional activities, and reduced PPARγ activity is independent of the protein level. B3 and E4 STHdh^Q7^ cells and 1A and 6L STHdh^Q111^ cells were transiently transfected with PPRE, CRE, or PGC-1α promoter luciferase reporter plasmids. The basal activities of PPRE (*A*), CRE (*B*), and PGC-1α promoter (*C*) reporters were dramatically reduced in both 1A and 6L STHdh^Q111^ cells. *n* = 3–4 Data shown are mean ± SE. *D*, PPARγ protein levels were variable in B3 and E4 STHdh^Q7^ cells and 1A and 6L STHdh^Q111^ cells, while the original STHdh^Q111^ cells exhibited lower levels of PPARγ than the original STHdh^Q7^ cells. Sixty micrograms of protein was run in each lane.

We previously showed that PPARγ signaling is impaired in STHdh^Q111^ cells [6]. To confirm that disturbance of PPARγ signaling was due to the presence of mHtt we measured PPARγ activity in additional striatal cell lines. Two new striatal cell lines expressing mHtt (1A and 6L) exhibited a substantial reduction in the basal activity of PPARγ compared to two new striatal cell lines expressing Htt (B3 and E4) (Fig. 3*A*). Furthermore, CRE basal activity and PGC-1α promoter activity were also significantly compromised in 1A and 6L STHdh^Q111^ cells, which is in agreement with previous reports (Fig. 3, *B* and *C*) [6], [12]. However, we found that PPARγ protein levels were variable in B3 and E4 while the original STHdh^Q111^ cells exhibited lower levels of PPARγ than the original STHdh^Q7^ cells (Fig. 3*D*). This result indicates that the repressed activity of PPARγ is independent of its expression level.

### Protective Effect of Rosiglitazone Is Due to the Specific Activation of PPARγ

**Figure 4:**
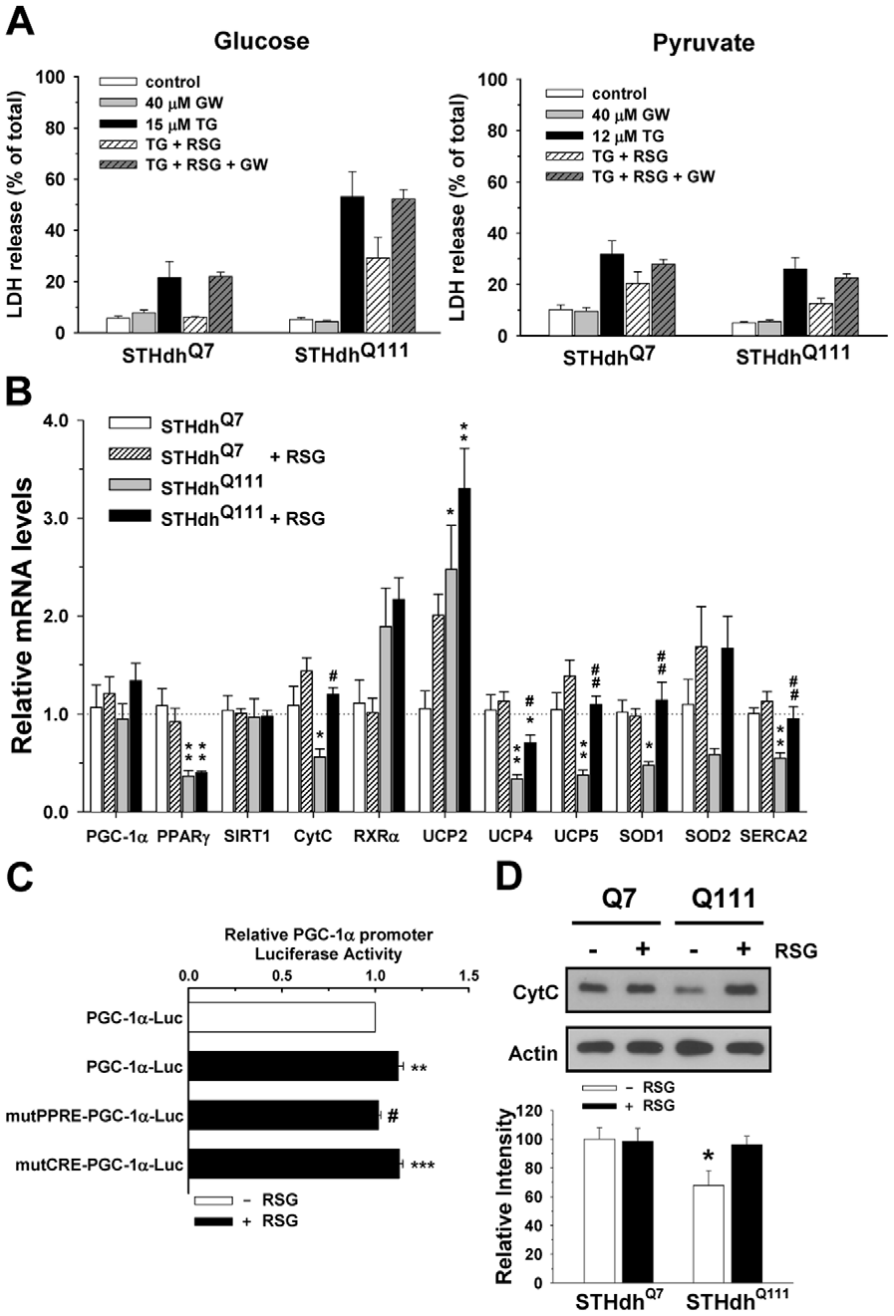
The protective effect of RSG is due to the specific activation of PPARγ. *A*, GW9662 (GW), a PPARγ antagonist, abolishes the protective effect of RSG on TG-induced cell death in the glucose and pyruvate conditions. *n* = 4–6. 40 µM GW9662 was added in the presence or absence of 20 µM RSG for 24 h prior to TG treatment. *B*, RSG increases the expression of genes involved in mitochondrial function (CytC, UCP2, UCP4, UCP5), calcium regulation (SERCA2), and ROS response (SOD1, SOD2), but does not change the expression of PPARγ and SIRT1. *n* = 4–6. * *P*<0.05, ** *P*<0.01, *** *P*<0.001 vs. control of STHdh^Q7^; ^#^ *P*<0.05, ^##^ *P*<0.01, ^###^ *P*<0.001 vs. control of each cell type. Statistical significance was determined by one-way ANOVA followed by Student-Newman-Keuls multiple comparisons test. *C*, PGC-1α promoter is slightly but significantly activated by RSG in STHdh^Q111^ cells. The mutation at PPRE but not CRE sites in PGC-1α promoter completely abolishes PGC-1α promoter activation induced by RSG treatment. *n* = 4–6. ** *P*<0.01, *** *P*<0.001 vs. control; ^#^ *P*<0.05 vs. RSG. *D*, RSG increases the protein level of CytC in STHdh^Q111^ but not STHdh^Q7^ cells. Data shown are mean ± SE.

To determine if the protective effects of RSG on TG-induced cell death were specifically due to PPARγ activation, we co-administered GW9662, a PPARγ antagonist, with RSG for 24 h prior to TG treatment. GW9662 completely abolished the protective effects of RSG on TG-induced cell death, suggesting that the protective effect of RSG stems from PPARγ activation (Fig. 4*A*). To test whether the reduced activity of PPARγ in striatal cells is sufficient to sensitize striatal cells to stressors in the glucose condition, shRNA for PPARγ was stably expressed in STHdh^Q7^ cells (Fig. S3). The stable expression of shRNA- PPARγ significantly reduced the activity of PPARγ in STHdh^Q7^ cells (Fig. S3A). However, cell death induced by treatment with H_2_O_2_ or TG was not significantly increased by the stable expression of shRNA- PPARγ in STHdh^Q7^ cells (Fig. S3B). This result suggests that reduced PPARγ activity is not the only causative factor in the sensitization of STHdh^Q111^ cells to stressors in the glucose condition.

We next examined how RSG treatment affected the expression of specific genes in STHdh^Q7^ and STHdh^Q111^ cells. Striatal cells were incubated with or without 24 h of RSG treatment in the glucose condition prior to collection. Quantitative RT-PCR was performed as described in materials and methods (Fig. 4*B*). STHdh^Q111^ cells show a significantly reduced expression of genes related to mitochondrial function [PPARγ, cytochrome C (CytC), uncoupling proteins (UCP4, UCP5)], calcium regulation [sarco(endo)plasmic reticulum Ca^2+^-ATPase 2 (SERCA2)], and ROS response [superoxide dismutase 1 (SOD1)]. SOD2 appears to be decreased in STHdh^Q111^ cells. Retinoid X receptor α (RXRα), an obligatory signaling partner of PPARγ, appears to be increased in STHdh^Q111^ cells, suggesting that the reduced activity of PPARγ is not attributable to RXRα expression. RSG significantly upregulated CytC, UCP4, UCP5, SOD1, and SERCA2 in STHdh^Q111^ but not STHdh^Q7^ cells. PPARγ, RXRα, and SIRT1 showed no change after RSG treatment in both cell types. UCP2 was significantly increased and appeared to be further induced by RSG treatment in STHdh^Q111^ cells. PGC-1α trended higher in both cell types after RSG treatment but the increase was not statistically significant. PGC-1α gene regulation in STHdh^Q111^ cells was further investigated using PGC-1α promoter luciferase reporter assay (Fig. 4*C*). RSG induced a slight but significant increase of PGC-1α promoter activity and mutation of the PPAR-response element (PPRE), but not the cAMP response element (CRE) site, in the PGC-1α promoter completely abolished RSG-induced activation. These results suggest that PGC-1α expression is likely upregulated by RSG treatment in STHdh^Q111^ cells. Immunoblot of CytC showed a significantly reduced level in STHdh^Q111^ cells compared to STHdh^Q7^ cells. RSG increased CytC expression in STHdh^Q111^ but not STHdh^Q7^ cells, confirming the quantitative RT-PCR data (Fig. 4, *B* and *D*).

### Metabolic Conditions Differentially Impact Mitochondrial Membrane Potential Loss (ΔΨm) in Response to Thapsigargin

**Figure 5:**
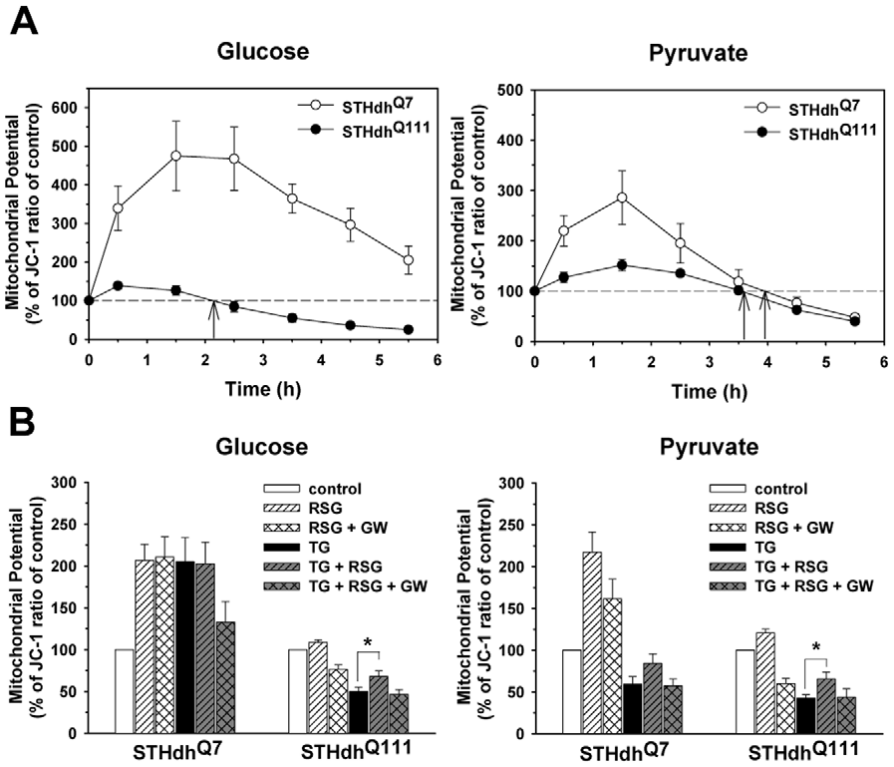
Metabolic conditions differentiate ΔΨm-loss in response to TG. *A*, In the glucose condition STHdh^Q111^ cells undergo ΔΨm-loss in response to TG at a significantly faster rate than STHdh^Q7^ cells. In the pyruvate condition, ΔΨm of both cell types shows similar kinetics in response to TG. Arrows indicate the point at which ΔΨm begins to drop below the baseline. *n* = 4. *B*, RSG slightly but significantly delays TG-induced ΔΨm-loss in glucose and pyruvate conditions. GW9662 abrogates the delayed ΔΨm-loss by RSG treatment. *n* = 5–9. Data shown are mean ± SE. * *P*<0.05.

ΔΨm is tightly linked with mitochondrial functions such as Ca^2+^ buffering, ATP synthesis, and cell death processes [28]. In order to understand how different metabolic conditions result in different patterns of TG-induced cell death, we monitored ΔΨm using JC-1 dye [29], [30]. Treatment with FCCP, a mitochondrial uncoupler, markedly reduced the JC-1 ratio, validating the response of JC-1 dye to ΔΨm (data not shown). TG treatment increased the ΔΨm at early time points in both cell types in both metabolic conditions. ΔΨm in STHdh^Q111^ cells began to drop 2 h after TG treatment in the glucose condition, while STHdh^Q7^ cells maintained ΔΨm above the base line until 6 h. TG treatment in the pyruvate condition resulted in similar kinetic responses in both cell types with the ΔΨm dropping below the base line after 4 h (Fig. 5*A*). These results suggest that the distinct kinetic responses in TG-induced ΔΨm-loss in the different metabolic conditions may be a contributing factor to the different cell death profiles. We next tested whether RSG affects TG-induced ΔΨm loss. ΔΨm was measured 5 h after adding TG (Fig. 5*B*). Treatment of STHdh^Q7^, but not STHdh^Q111^ cells, with RSG dramatically increased the basal level of ΔΨm in both glucose and pyruvate conditions. RSG treatment slightly but significantly delayed TG-induced ΔΨm-loss of STHdh^Q111^ cells, an effect that was completely abrogated by GW9662 treatment, indicating the specific activation of PPARγ by RSG treatment.

### PPARγ Activation Reduces Caspase Activation Induced by Thapsigargin

**Figure 6:**
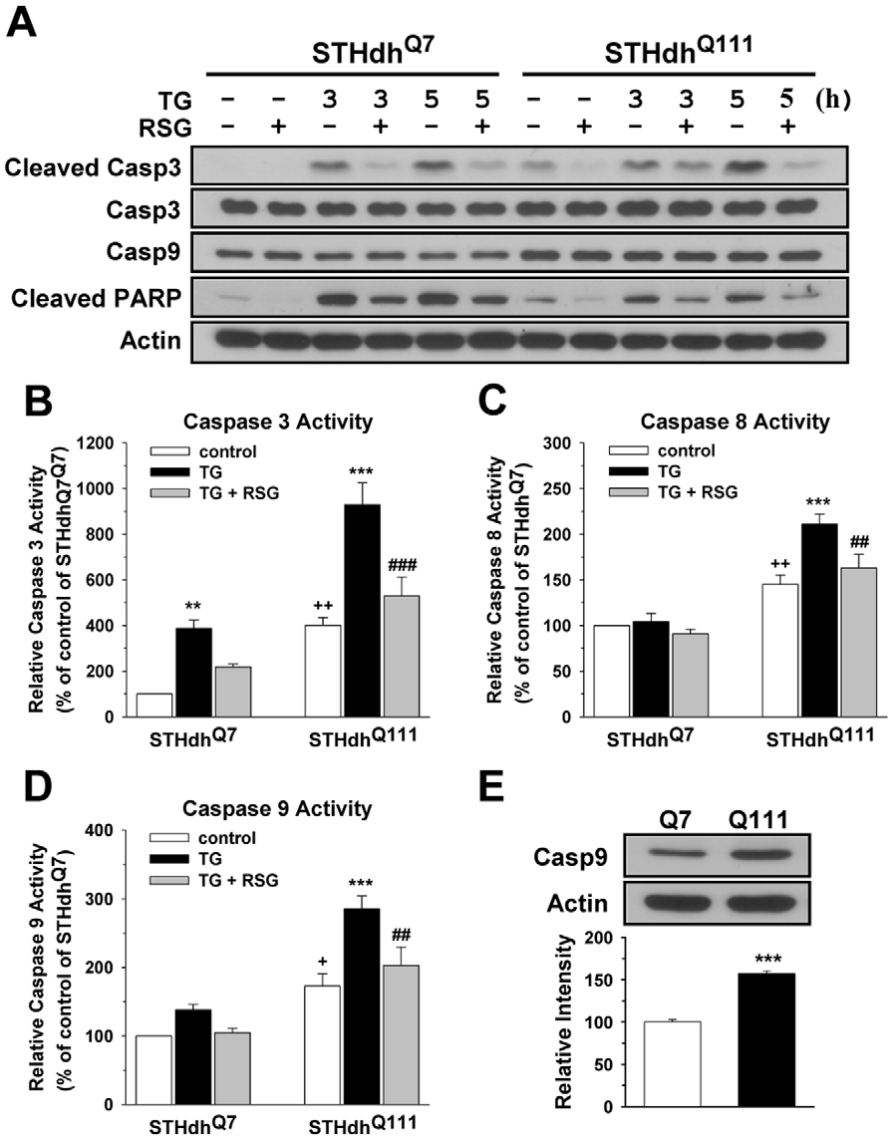
RSG reduces TG-induced caspase activation. *A*, TG induces the cleaved forms of caspase 3 and PARP in both cell types, responses that were reduced by RSG. *B*, TG increases caspase 3 activity of STHdh^Q111^ cells to a significantly greater extent compared to STHdh^Q7^ cells, a response that was significantly attenuated by RSG. Basal activity of caspase 3 in STHdh^Q111^ cells was significantly higher compared to STHdh^Q7^ cells. *n* = 5. *C*, TG increases caspase 8 activity in STHdh^Q111^ but not STHdh^Q7^ cells, a response that was significantly attenuated by RSG. Basal activity of caspase 8 of STHdh^Q111^ cells is significantly higher compared to STHdh^Q7^ cells. *n* = 5. *D*, TG increases caspase 9 activity in STHdh^Q111^ but not STHdh^Q7^ cells, a response that was significantly attenuated by RSG. Basal activity (*D*) and basal level (*E*) of caspase 9 in STHdh^Q111^ cells are significantly higher compared to STHdh^Q7^ cells. *n* = 4–5. Data shown are mean ± SE. ** *P*<0.01, *** *P*<0.001 vs. control of each cell type; ^##^ *P*<0.01, ^###^ *P*<0.001 vs. TG of each cell type; ^+^ *P*<0.05, ^++^ *P*<0.01 vs. control of STHdh^Q7^. Statistical significance was determined by one-way ANOVA followed by Student-Newman-Keuls multiple comparisons test (*B*, *C*, and *D*) or by Student's *t* test (*E*).

Next, since caspase activation plays a pivotal role in cell death processes, we tested whether caspase activation is involved in TG-induced cell death and if RSG attenuates caspase activation. TG treatment resulted in the formation of cleaved caspase 3, an active form of caspase 3, after 3 h in both cell types in the glucose condition (Fig. 6*A*). RSG dramatically reduced the level of cleaved caspase 3 in both cell types. Interestingly, the basal level of cleaved caspase 3 in STHdh^Q111^ cells is much higher compared to STHdh^Q7^ cells and substantially decreased by RSG treatment, while the total level of caspase 3 is similar between two cell types. The basal level of caspase 9 in STHdh^Q111^ cells was higher than in STHdh^Q7^ cells (Fig. 6*E*). The total levels of caspase 3 and 9 were not changed in response to TG or by RSG treatment. TG strongly induced cleaved PARP (poly ADP-ribose polymerase), another indicator of caspase 3 activation, and RSG reduced the level of cleaved PARP in both cell types. Although these results indicate that RSG ameliorates TG-induced cell death in part by attenuating caspase activation, these results do not explain why TG-induced cell death is significantly greater in STHdh^Q111^ cells. Because immunoblot based assays do not represent the real activity of caspases, we next measured the activity of three different caspases in response to TG (Fig. 6, *B*–*D*). TG significantly increased the activities of caspase 3 and 9 in STHdh^Q111^ cells, while STHdh^Q7^ cells only exhibited increased activity of caspase 3 and to a much lesser extent than STHdh^Q111^ cells. In addition, caspase 8 activity was measured since TG increases the activity of caspase 8 [31], [32] and caspase 8 is implicated in HD [33], [34], [35]. Caspase 8 activity in STHdh^Q111^, but not STHdh^Q7^ cells, was significantly increased by TG treatment. RSG significantly diminished TG-induced activation of these caspases in STHdh^Q111^ cells. These results suggest that the greater cell death in STHdh^Q111^ cells by TG treatment may be due to the greater activation of caspases which is reduced by RSG. Furthermore, STHdh^Q111^ cells display higher basal activities of caspase 3, 8, and 9, which is in line with the immunoblot results. The higher basal activities of caspases may also contribute to the greater sensitivity of STHdh^Q111^ cells to the stressors.

### Stable Expression of Constitutively Active PPARγ Significantly Attenuates Cell Death Induced by H_2_O_2_ or Thapsigargin

**Figure 7:**
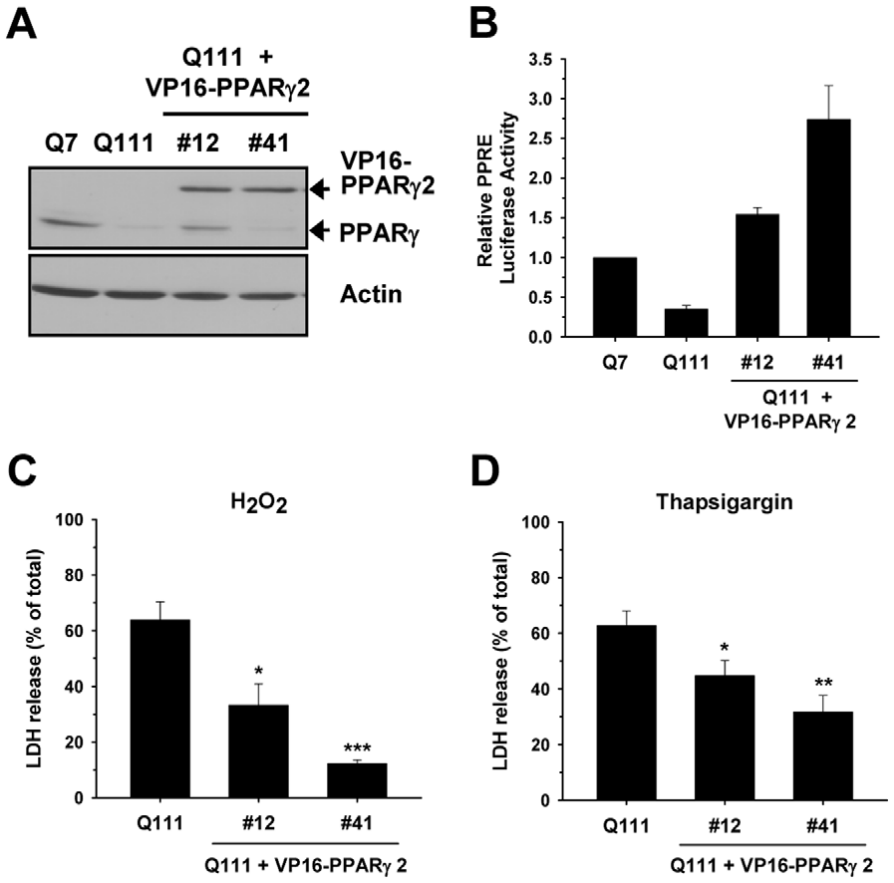
Stable expression of constitutively active PPARγ2, VP16- PPARγ2, in STHdh^Q111^ cells significantly attenuates cell death induced by H_2_O_2_ and TG. *A*, VP16-PPARγ2 is stably expressed in STHdh^Q111^ cells. #12 and #41 clonal STHdh^Q111^ cells were individually selected and used for experiments. *B*, Stable expression of VP16- PPARγ2 in STHdh^Q111^ cells significantly increases PPARγ activity in STHdh^Q111^ cells. *n* = 3. Stable expression of VP16- PPARγ2 in STHdh^Q111^ cells significantly diminishes cell death in response to 0.6 mM H_2_O_2_ (*C*) and by 12 µM TG (*D*) in the glucose condition. *n* = 4–6. The efficiency to reduce cell death seems to be proportional to the PPARγ activity. Data shown are mean ± SE. * *P*<0.05, ** *P*<0.01, *** *P*<0.001 vs. vehicle control.

It has been shown that RSG may affect signaling pathways independent of PPARγ pathway [36]. Therefore, we tested whether the protective effect of RSG mainly results from PPARγ activation. We established STHdh^Q111^ cells stably expressing constitutively active PPARγ, V16- PPARγ2 (Fig. 7*A*). Stable expression of VP16- PPARγ2 significantly increased PPARγ activity at the basal level (Fig. 7*B*). #41 cell line exhibited greater PPARγ activity than #12 cell line, although the expression levels of VP16- PPARγ2 were similar between two cell lines. Importantly, both #12 and #41 cell lines exhibited the greater activity of PPARγ compared to STHdh^Q7^ cells and showed significantly reduced cell death in response to H_2_O_2_ or TG in a PPARγ activity dependent manner (Fig. 7, *C* and *D*). These results indicate that PPARγ activation plays a pivotal role in the protective effect of RSG.

### Metabolic Condition Differentiates Superoxide/ROS Generation in Response to Thapsigargin

**Figure 8:**
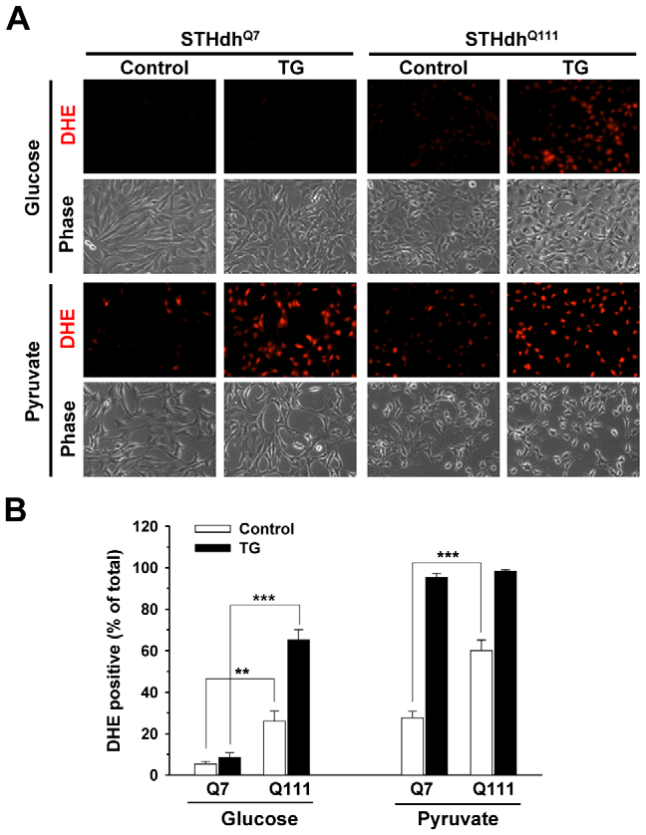
Metabolic conditions differentially affect superoxide/ROS generation. *A*, In the glucose condition TG-induced superoxide/ROS generation in STHdh^Q111^ cells was greater compared to STHdh^Q7^ cells, while in the pyruvate condition TG induced superoxide/ROS generation to a similar extent in both cell types. *B*, The percentage of DHE positive cells was calculated. Quantitative data shows the differential effects of metabolic conditions on TG-induced superoxide/ROS generation as shown in *A*. *n* = 6. STHdh^Q111^ cells display significantly higher basal levels of superoxide/ROS compared to STHdh^Q7^ cells. Data shown are mean ± SE. ** *P*<0.01, *** *P*<0.001.

We previously showed that TG induced a greater generation of ROS in STHdh^Q111^ cells compared to STHdh^Q7^ cells [6]. Superoxide can be generated in mitochondria and cytosol and contribute to cell death/stress signaling pathways. Hence we measured superoxide levels in different metabolic conditions using dihydroethidium (DHE), a commonly used dye for superoxide detection, although it has been suggested that DHE may be oxidized by other ROS [37]. Interestingly, different metabolic conditions resulted in distinct patterns of superoxide/ROS production (Fig. 8*A*). STHdh^Q111^ cells show higher basal levels of superoxide/ROS in both glucose and pyruvate conditions. TG induced a substantial amount of superoxide/ROS in STHdh^Q111^ but not STHdh^Q7^ cells in the glucose condition, while in the pyruvate condition TG significantly increased superoxide/ROS in both cell types to a similar extent (Fig. 8, *A* and *B*). This result suggests that the distinct cell death patterns between the two different metabolic conditions in response to TG maybe in part related to superoxide/ROS generation.

### PPARγ Activation Significantly Reduces Superoxide/ROS Generation

**Figure 9:**
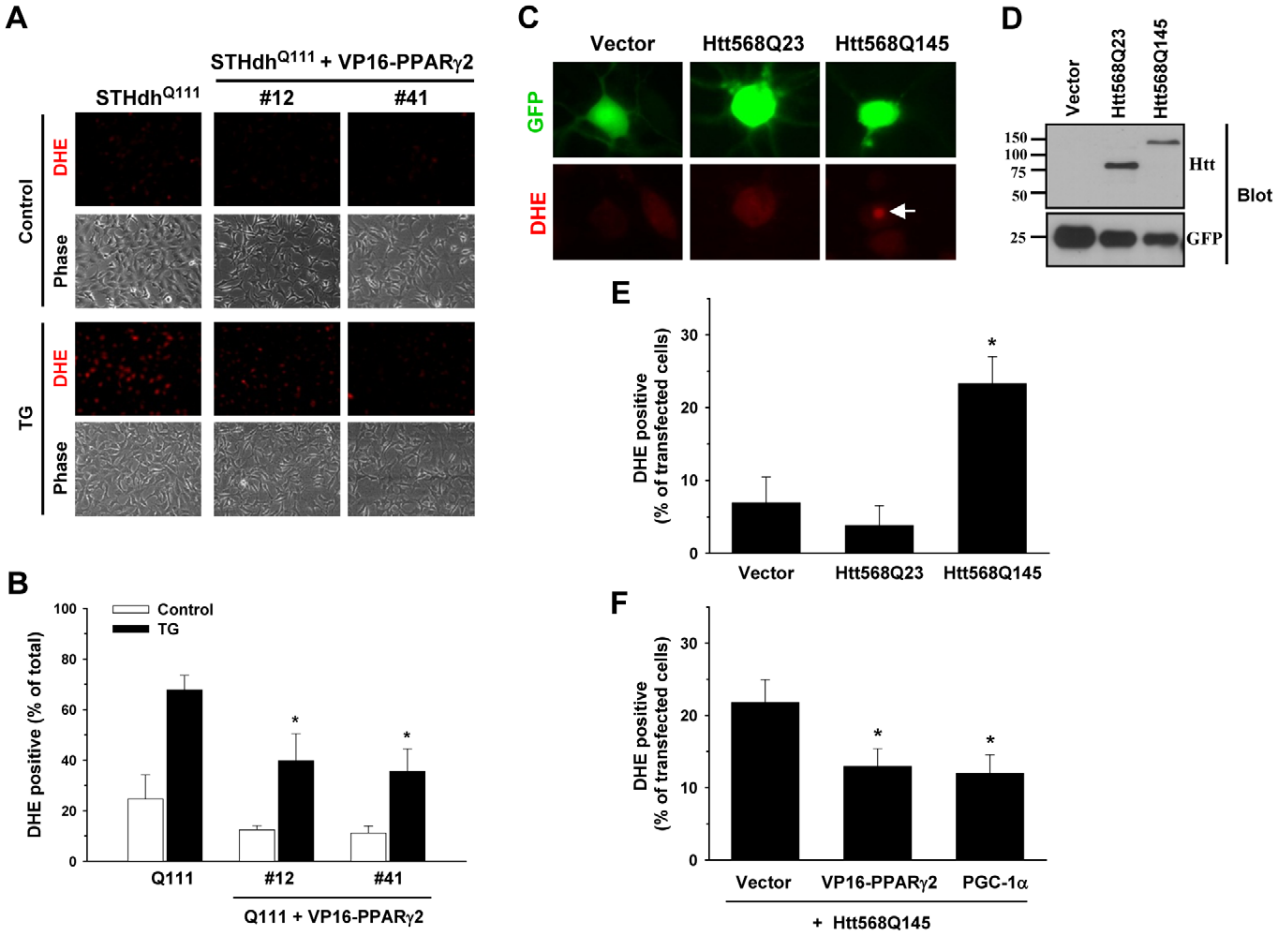
PPARγ activation significantly reduces superoxide/ROS generation. *A* and *B*, Stable expression of VP16- PPARγ2 in STHdh^Q111^ cells significantly reduces superoxide/ROS generation in response to TG. *A*, STHdh^Q111^ cells and #12 and #41 STHdh^Q111^ cells stably expressing VP16- PPARγ2 were treated with 12 µM TG for 1 h after adding 5 µM DHE for 10–15 min. Both cell lines stably expressing VP16- PPARγ2 show significantly lower level of superoxide/ROS generation than STHdh^Q111^ cells. *B*, Quantitative results show significantly reduced DHE positive cells in response to TG. *n* = 5. * *P*<0.05 vs. TG of STHdh^Q111^. *C*–*F*, Mutant huntingtin increases superoxide production, an effect that is significantly reduced by PPARγ activation in the primary rat cortical neuronal cells. *C*, Primary rat cortical neurons were transfected with vector, wild type Htt (Htt568Q23), or mHtt (Htt568Q145) on DIV 7. Since all three plasmids contain IRES-GFP, GFP positive neurons were monitored for DHE staining on DIV 13. DHE nucleus staining was observed more frequently in neurons transfected with Htt568Q145. Arrow indicates DHE stained nucleus. *D*, Vector, Htt568Q23, and Htt568Q145 were transfected in HEK cells and the expression of wild type Htt and mHtt were verified by immunoblotting. Htt568Q145 migrates much slower than Htt568Q23 due to the extensively long track of poly glutamine. *E*, DHE positive neurons were counted from all of transfected neurons. Htt568Q145 transfection significantly increases the frequency of DHE positive neurons compared to vector or Htt568Q23 transfection. *n* = 4. * *P*<0.05 vs. vector. *F*, VP16- PPARγ2 or PGC-1α cotransfection significantly reduces the frequency of DHE positive neurons transfected with Htt568Q145. *n* = 7. * *P*<0.05 vs. vector and Htt568Q145. Data shown are mean ± SE.

PPARγ plays an important role in regulating defense mechanisms against oxidative stress [38]. Therefore, we investigated whether PPARγ activation ameliorates superoxide/ROS production in HD cell models. We tested STHdh^Q111^ cells stably expressing VP16- PPARγ2. The stable expression of VP16- PPARγ2 led to pronounced reductions in TG-induced superoxide/ROS generation in STHdh^Q111^ cells in the glucose condition (Fig. 9*A*). The quantitative data shows that TG-induced superoxide/ROS production is significantly attenuated in two stable cell lines expressing VP16- PPARγ2 (Fig. 9*B*), suggesting that the protective effect of PPARγ activation on TG-induced cell death involves the regulation of oxidative responses including superoxide. We next investigated whether mHtt expression enhances superoxide/ROS production in primary neurons. Rat primary cortical neurons were transfected with vector, Htt (Htt568Q23), or mHtt (Htt568Q145) on DIV 7 and superoxide/ROS generation was measured 6 days later (Fig. 9, *C*–*F*). The expression of Htt568Q23 and Htt568Q145 were verified by immunoblot of HEK cells after transfection (Fig. 9*D*). Primary neurons expressing Htt568Q145 exhibited a higher frequency of DHE positive cells than those transfected with vector or Htt568Q23 (Fig. 9, *C* and *E*). Next, we tested whether cotransfection of VP16- PPARγ2 with Htt568Q145 decreases the percentage of DHE positive neurons. The frequency of DHE positive neurons was significantly reduced by VP16- PPARγ2 expression compared to cotransfection of an empty vector with Htt568Q145 (Fig. 9*F*). For comparison, PGC-1α was cotransfected with Htt568Q145, which also resulted in a significant reduction in the percentage of DHE positive neurons (Fig. 9*F*). These results suggest that mHtt increases oxidative stress, presumably including superoxide, and that the activation of PPARγ/PGC-1α may be a promising target to protect neurons from increased oxidative stress.

## Discussion

Numerous studies have suggested that bioenergetic impairment is an important contributing factor to HD pathogenesis [3], [4], [5]. These include the finding that the activity of the pyruvate dehydrogenase complex (PDH), which links glycolysis and TCA cycle/Oxphos, is significantly reduced in brain of HD patients [39], [40]. These studies led us to hypothesize that different metabolic conditions may result in alterations in the susceptibility of neuronal cells expressing mHtt to different stressors. Our initial prediction was that in the pyruvate condition, where cellular bioenergetics is predominantly dependent on mitochondrial Oxphos, greater differences in cell death between STHdh^Q7^ and STHdh^Q111^ cells would be observed. Unexpectedly, it was in the glucose condition in which the greater differences between two cell types were observed, with the extent of cell death induced by H_2_O_2_ or TG being significantly greater in STHdh^Q111^ cells. Our initial hypothesis was that due to the mitochondrial deficits in the STHdh^Q111^ cells, specifically deficiencies in ATP production [20], [21], greater differences in stress-induced cell death would be observed in the pyruvate condition. However in retrospect, it is not surprising that the greater differences were observed in the glucose condition. First, deficits in PDH have been reported in HD cases [39], [40], as well as in HD mouse models [41], which in combination with other defects in mitochondria bioenergetics, may make STHdh^Q111^ cells more reliant on glycolysis for ATP production, and thus more vulnerable to stress in the glucose condition. Second, we assumed that a subtle energy deficiency would be a pivotal contributor to cell death in response to H_2_O_2_ or TG, however it is not unreasonable that other variables are more important in determining cell death outcomes. Taken together, our study provides new insight for understanding the interrelationship between bioenergetic disturbances and the pathogenesis in HD.

Increased caspase activation has been associated with HD pathogenesis [33], [34], [35]. Our results demonstrate that the protective mechanisms of PPARγ activation against TG-induced cell death involve a slight delay of ΔΨm loss (Fig. 5) and inhibition of caspase activation (Fig. 6). The basal activities of three caspases (3, 8, and 9) were higher in STHdh^Q111^ cells compared to STHdh^Q7^ cells, rendering mHtt-expressing striatal cells more vulnerable to various stressors. A delay of ΔΨm-loss could be a contributing factor to the inhibitory effect of RSG on caspase activation. Since in the glucose condition STHdh^Q111^ cells exhibited much faster kinetics of TG-induced ΔΨm-loss than STHdh^Q7^ cells, we also examined major components of mPTP such as cyclophilin D (CypD) and voltage-dependent anion channel (VDAC) (Fig. S4). In general, the higher expression of CypD supposedly increases the probability of mPTP opening [42], [43], [44]. However, the basal expression of CypD was lower at both the mRNA and protein level in STHdh^Q111^ cells than STHdh^Q7^ cells, while the expression of VDAC appeared to be equivalent in the two cell types. In addition, RSG treatment did not alter the expression of CypD or VDAC (Fig. S4 *C*). These results suggest that the protective effect of RSG and the higher susceptibility of STHdh^Q111^ cells to TG may not be related to mPTP. Similarly, a recent study using R6/2 mice crossed with CypD knockout mice demonstrated that the deletion of CypD in the R6/2 mice resulted in enhanced mitochondrial Ca^2+^ buffering but did not show any improvement in the pathogenic symptoms or a delay in disease progression [45]. Further, RSG has been reported to protect cells against stresses by upregulation of PPARγ and Bcl-2, an anti-apoptotic protein that has been shown to be implicated with HD [14], [46]. To test whether the protective effect of RSG could be due to an increase of Bcl-2 and/or PPARγ, we monitored the expression of Bcl-2 and PPARγ in both cell types in the presence or absence of RSG. STHdh^Q111^ cells show significantly lower levels of Bcl-2 and PPARγ in the basal condition (Fig. S4, *A* and *C*). RSG treatment did not affect the expression of Bcl-2 or PPARγ (Fig. S4 *C*), indicating that the protective effect of RSG is not due to the upregulation of Bcl-2 or PPARγ.

TG-induced cell death can be mediated by endoplasmic reticulum (ER) stress and unfolded protein response (UPR) [47], [48]. UPR induces the expression of the molecular chaperone BiP/GRP78 and activates the ER-resident caspase-12 through processing by calpain and caspase. TG did not induce the expression of BiP/GRP78 or increase cleaved form of caspase 12 in two cell types. RSG did not alter BiP/GRP78 expression or caspase 12 activation in the presence or absence of TG (Fig. S5). This result suggests that the UPR is probably not a major contributor to TG-induced cell death in these experimental conditions, and the protective effect of RSG does not result from modulation of UPR. However, interestingly, the pro and active forms of caspase 12 are much higher in STHdh^Q111^ cells compared to STHdh^Q7^ cells. Taken together with the higher basal activity of caspase 3, 8, and 9, this result suggests that the higher levels of the activity and/or expression of caspases may render STHdh^Q111^ cells more susceptible to cellular or exogenous stresses.

mHtt has been proposed to impair mitochondrial function by direct interaction [49], [50] as well as indirectly by transcriptional dysregulation including PGC-1α signaling [12]. PGC-1α and PPARγ play important roles in mitochondrial biogenesis and detoxification of ROS. We previously demonstrated that PPARγ signaling was severely compromised in STHdh^Q111^ cells [6]. Consistent with the repressed activity of PGC-1α/ PPARγ the expression of genes under the control of PGC-1α/ PPARγ such as CytC and SOD1 were significantly downregulated and RSG rescued reduced expression of those genes in STHdh^Q111^ cells (Fig. 4*B*). Despite the compromised signaling of PPARγ, the basal level of UCP2 was upregulated in STHdh^Q111^ cells and further induced by RSG treatment. Increased UCP2 at the basal level could be due to genetic compensation by other PPAR family such as PPARα and PPARβ, since UCP2 can be upregulated by PPARα and PPARβ [51], [52] or a response to increased superoxide level in STHdh^Q111^ cells as reported previously that superoxide upregulates UCP2 level [53], [54]. Furthermore, it has been suggested that superoxide activates UCP2 [55], [56]. Increased or activated UCP2 may act as a protective mechanism in the STHdh^Q111^ cells [57]. The pyruvate condition resulted in a subtle change in gene expression profiles compared to the glucose condition (Fig. S6). The expression profiles of PGC-1α, PPARγ, SIRT1, UCP2, and UCP4 in the pyruvate condition were similar to those shown in the glucose condition. However, in the pyruvate condition the two cell lines exhibited similar levels of CytC, UCP5, SOD1, SOD2, and SERCA2 which were shown to be decreased in STHdh^Q111^ cells in the glucose condition, suggesting that different metabolic environments can alter gene regulation. Since the levels of SOD1 and SOD2 were reduced in STHdh^Q111^ cells in the glucose condition but not in the pyruvate condition, we investigated if superoxide/ROS generation in two cell types may differ in different metabolic conditions. STHdh^Q111^ cells produced more superoxide/ROS at the basal level and in response to TG in the glucose condition compared to STHdh^Q7^ cells, while in the pyruvate condition similar profiles of superoxide/ROS generation were observed in both cell types (Fig. 8). The profiles of TG-induced superoxide production in the two cell types in different metabolic conditions are similar to those of TG-induced cell death, suggesting that superoxide/ROS is likely to be a crucial mediator of TG-induced cell death. In addition, a large body of evidence provides evidence that PPARγ activation reduces superoxide/ROS generation in various models [38], [58], [59].

Oxidative stress has been proposed as one of key components in the pathogenesis of HD [8], [9], [10]. Recent studies using proteomic approaches identified proteins modified by oxidative stress in human HD samples and R6/2 mice and the activities of oxidized proteins were severely compromised [9], [10]. Interestingly, many identified proteins are involved in glycolysis or mitochondrial metabolism, suggesting that oxidative stress could lead to metabolic disturbances and neuronal dysfunction [9], [10]. We found that in the glucose condition STHdh^Q111^ cells showed reduced expression of genes involved in ROS response at least in part due to the repressed signaling pathways of PPARγ/PGC-1α and produced higher levels of superoxide/ROS in basal condition than STHdh^Q7^ cells. We postulate that mHtt interferes with transcriptional processes, leading to disruption in the expression of genes involved in bioenergetics and ROS response, in turn resulting in impaired metabolism and enhanced ROS. Increased oxidative stress may have an impact on transcriptional processes. For example, the promoter of SQSTM1/p62 exhibited oxidative damage in samples from HD samples, resulting in decreased expression of p62 [60].

In summary, we demonstrate for the first time that different metabolic states result in surprisingly differential cellular sensitivities to stressors in the context of HD. We also directly demonstrate that PPARγ activation significantly attenuated superoxide/ROS production and cell death in response to stressors in striatal precursor cells and primary cortical neurons expressing mHtt. This study provides new important insights into a cycling feed forward mechanism in HD involving transcriptional dysregulation, oxidative stress, and metabolic impairment and proposes PPARγ as a potential target for a therapeutic strategy in HD.

## Materials and Methods

### Ethics Statement

All animal protocols have been approved by the UCAR at the University of Rochester (UCAR#2007-023R).

### Materials

Rosiglitazone (RSG), GW9662, rolipram (RP), thapsigargin (TG), and caspase substrates were purchased from Alexis. dihydroethidium (DHE) was purchased from Invitrogen. All other chemicals were purchased from Sigma, if not otherwise indicated.

### Plasmid Constructs

PPRE×3-TK-Luc and human PGC-1α promoter-Luc were obtained from Addgene [61], [62]. The human PPARγ and PGC-1α constructs were purchased from OriGene. mutPPRE- PGC-1α promoter-Luc and mutCRE- PGC-1α promoter-Luc were kindly provided from Dr. Francesc Villarroya [63]. pCRE-Luc was purchased from Clontech. VP16- PPARγ2 construct was a gift from Dr. Mitchell Lazar. To make VP16- PPARγ2-pHM6/PUR, VP16- PPARγ2 was amplified by PCR with primers containing MfeI/NotI sites and subcloned into EcoRI/NotI sites of pHM6/PUR. pHM6/PUR was made by inserting the blunt ended puromycin resistance gene at the PsiI site of pHM6 (Roche). The full length wild type human Htt DNA with 23 polyQ, pRc/CMV-HDFLQ23, was a gift from Dr. Christopher Ross and the full length mutant human Htt DNA with 145 polyQ was obtained from CHDI. To make constructs of truncated huntingtin with 568 amino acids, Htt568Q23 and Htt568Q145, each fragment of the huntingtin cDNA of Htt568Q23 and Htt568Q145 was generated by PCR with primers including BamHI/EcoRI sites and the respective full length huntingtin DNA was used as a template. PCR products were digested with BamHI and EcoRI and then subcloned into FIGB vector derived from FG12 vector [64]. FIGB contains IRES-GFP so that transfected cells can be identified.

### Cell Culture and Different Metabolic Media

The immortalized striatal precursor cell lines, STHdh^Q7^ (the original one, B3, E4) and STHdh^Q111^ (the original one, 1A, 6L), made from striatal primordia of E14 mouse embryos expressing Htt with 7 polyQ or mHtt with 111 polyQ were kindly provided by Dr. Marcy MacDonald [65]. Cells were cultured in DMEM containing 25 mM glucose and 4 mM glutamine (Invitrogen) supplemented with 4% fetal bovine serum (FBS, HyClone) and 4% bovine growth serum (BGS, HyClone), and 100 units/ml penicillin and 100 µg/ml streptomycin (Invitrogen) in the incubator at 33°C containing 5% CO_2_. For the two different metabolic conditions, 24 h after plating, the medium was completely changed as previously described with modification [23]. Glucose medium consists of DMEM containing 25 mM glucose and 4 mM glutamine supplemented with 10 mM Hepes and 2% dialyzed FBS (HyClone). Pyruvate medium consists of DMEM containing 4 mM glutamine without glucose (Invitrogen) supplemented with 5 mM galactose, 10 mM Hepes, 4 mM sodium pyruvate, and 2% dialyzed FBS. The dialyzed FBS is necessary to rule out any exogenous metabolic contributions.

### Generation of Stable Cell Lines

STHdh^Q111^ cells were transfected with VP16- PPARγ2-pHM6/PUR using Lipofectamine 2000 (Invitrogen). STHdh^Q111^ cells stably expressing VP16- PPARγ2 were selected by treatment with 2.5 µg/ml puromycin. Approximately 3 weeks later, individual colonies were picked, amplified, and tested for the expression and the activity of VP16- PPARγ2. #12 and #41 clonal cells were selected and used for study.

### Primary Cortical Neuronal Culture

Primary cortical neuronal culture from rat embryos was prepared as described previously with modification [66]. In brief, whole brains were removed from E17-18 rats. The cortices were then dissected, treated with 0.05% trypsin at 37°C for 30 min, and gently triturated with a fire polished glass Pasteur pipette. Dissociated cells were plated onto glass-coverslips coated with 40 µg/ml poly-D-lysine (Millipore) with Minimum Essential Media (MEM, Invitrogen) containing 25 mM Hepes and GlutaMAX equivalent to 2 mM glutamine supplemented with 5% FBS in the incubator at 37°C containing 5% CO_2_. Five hours after plating, medium was replaced with Neurobasal medium (NBM, Invitrogen) supplemented with 0.4 mM glutamine and B27 (Invitrogen). Every 3 days, half of the medium was removed, collected as conditioned NBM, and replenished with the complete NBM.

### Lactate Dehydrogenase (LDH) Release Assay

LDH release was measured using LDH release assay kit (Roche) as an assessment of cell death. Cells were plated in 48 well plates and were ∼80–90% confluent after 24 h. Media were replaced with different metabolic media in the absence or presence of 20 µM RSG for 24 h prior to TG or H_2_O_2_ treatment. Twelve hours after TG or H_2_O_2_ treatment, LDH release was measured following the manufacturer's instructions.

### Resazurin Assay

The cell viability was determined by monitoring conversion of resazurin into a fluorescent product resorufin using CellTiter-Blue Cell Viability Assay kit (Promega). Twenty four hours after plating cells in 24 well plates, media were replaced with different metabolic media. Next day cells were treated with TG or H_2_O_2_. Resazurin solution was added to the media after 6 h according to manufacturer's instructions. Two hour after incubation, resorufin fluorescence was measured using Synergy HT plate reader (BioTek) with excitation at 540 nm and emission at 590 nm. Results are presented as a percent of control cells.

### RNA Isolation, Reverse Transcription, and Real-time PCR

Cells were plated followed by incubation in either glucose or pyruvate media. After 24 h, total RNA was extracted using TRIzol (Invitrogen) according to the manufacturer's instruction. Extracted total RNA was treated with RNase free- DNaseI Amplification Grade (Invitrogen) to remove contaminating DNA. Two micrograms of total RNA was reverse transcribed using SuperScriptIII reverse transcriptase and random hexamers (Invitrogen). The reaction mixture was diluted with 680 µl of DEPC-treated H_2_O. The PCR reaction was prepared in triplicate containing 10 µl of diluted cDNA, 2.5 µl of 2.5 µM primer mixture (forward and reverse), and 12.5 µl of SYBR GreenER qPCR SuperMix (Invitrogen) in 96 well optical PCR reaction plate (Bio-Rad). PCR reactions were performed in MyiQ real-time PCR system (BioRad). Amplification conditions consisted of an initial hot start at 95°C for 10 min followed by amplification of 45 cycles (95°C for 15 s, 60°C for 20 s, and 72°C for 40 s). Melting curve analysis was performed immediately after amplification. The relative amount of mRNAs was calculated by using the ΔΔCt (Ct, threshold cycle) method. The Ct value of TATA binding protein (TBP) was used for normalization. The sequences of primers are shown in Table 1.

**Table 1:** Primers used for real-time PCR.

GENES	FORWARD PRIMER	REVERSE PRIMER	ACCESSION No.
PGC-1α	GAAGTGGTGTAGCGACCAATC	AATGAGGGCAATCCGTCTTCA	NM_008904
PPARγ	GGAAGACCACTCGCATTCCTT	TCGCACTTTGGTATTCTTGGAG	NM_011146
SIRT1	GACGATGACAGAACGTCACAC	TTCGAGGATCGGTGCCAATCA	NM_019812
CytC	CCAAATCTCCACGGTCTGTTC	ATCAGGGTATCCTCTCCCCAG	NM_007808
RXRα	TGACATGCAGATGGACAAGACGGA	TGCAGTACGCTTCTAGTGACGCAT	NM_011305
UCP2	AGCCTACAAGACCATTGCACGAGA	ATAGGTCACCAGCTCAGCACAGTT	NM_011671
UCP4	TGCAAATGGAAGGGAAACGCAGAC	AGCGCTGCTCTCTGAATATTGGGT	AB106930
UCP5	AACCCTGTGGATGTGGTGAGAACT	TGATGTTCCAGGGTCCAAGTCGAA	AF155812
SOD1	GGTGTGGCCAATGTGTCCATTGAA	TACTGCGCAATCCCAATCACTCCA	NM_011434
SOD2	TTAAGGAGAAGCTGACAGCCGTGT	TGTTGTTCCTTGCAATGGGTCCTG	NM_013671
SERCA2	TGGGCAAAGTGTATCGACAGGACA	GCAGGAACTTTGTCACCAACAGCA	AJ223584
CypD	AATGGGACAGGTGGCGAAAGTA	CACATGTTTCCCGTCCAGATGA	NM_026352
TBP	ATGCCTTACGGCACAGGACTTACT	AGTTGCTACTGCCTGCTGTTGTTG	NM_013684

PGC-1α, peroxisome proliferator-activated receptor γ (PPARγ) coactivator-1α; SIRT1, sirtuin 1; CytC, cytochrome C; RXRα, retinoid X receptor α; UCP, uncoupling protein; SOD, superoxide dismutase; SERCA2, sarco(endo)plasmic reticulum Ca^2+^-ATPase 2; CypD, cyclophilin D; TBP, TATA binding protein.

### Western Blot Analysis

Cells were washed with ice-cold phosphate-buffered saline (PBS) and lysed with modified RIPA buffer (50 mM Tris-HCl pH 7.4, 150 mM NaCl, 1% Triton X-100, 0.4% SDS, 0.2% sodium deoxycholate, 5% glycerol, 1 mM EDTA, 20 mM NaF, 2 mM Na_3_VO_4_) containing protease inhibitors (1 mM PMSF, 10 µg/ml leupeptin, 10 µg/ml aprotinin, 10 µg/ml pepstatin). The lysates were sonicated, cleared by centrifugation, and assayed to determine protein concentration using BCA assay (Pierce Biotechnology). Proteins (10–100 µg) were separated by SDS-PAGE and transferred to nitrocellulose membrane. The membrane was blocked with 5% skim milk in Tris-buffered saline containing 0.05% Tween 20 (TBST), and incubated with the specific antibodies in TBST containing 2% BSA or skim milk at 4°C overnight. Antibodies for PPARγ (1∶500), caspase 3 (1∶1,000), cleaved caspase 3 (1∶1,000), caspase 9 (1∶1,000), caspase 12 (1∶1,000), cleaved PARP (1∶1,000), and VDAC (1∶2,000) were acquired from Cell Signaling Technology, antibodies for huntingtin (1∶1,000) and actin (1∶50,000) were obtained from Chemicon, the antibody for cytochrome C (1∶2,000) was purchased from BD Biosciences Pharmingen, the antibody for Bip/GRP78 (1∶2,000) was obtained from Stressgen, the antibody for cyclophilin D (1∶5,000) was purchased from Calbiochem, the antibody for α-tubulin (1∶2,000) was purchased from Santa Cruz Biotechnology, and the antibody for Bcl-2 (1∶1,000) was purchased from Sigma. After washing three times, HRP-conjugated secondary antibody (1∶3000) in TBST containing 5% skim milk was applied and the blot was visualized by chemiluminescence. The intensity of immunoreactive bands was quantified by using Image J software.

### Dual Luciferase Reporter Assays

Cells were plated in 24 well plate. The next day, a reporter plasmid (3×PPRE-Luc, PGC-1α promoter-Luc, mutPPRE-PGC-1α promoter-Luc, mutCRE-PGC-1α promoter-Luc, or CRE-Luc) and a normalizing plasmid phRL-TK (Promega) were transfected using Lipofectamine 2000. The next day, cells were treated with vehicle control or drug. After 16 h, cells were lysed with Passive lysis buffer (Promega), and the reporter activity was measured using the Dual-Luciferase Reporter Assay System (Promega). The reporter activity from Firefly luciferase was normalized with the Renilla luciferase activity.

### Caspase Activity Assay

Cells were treated with 20 µM RSG for 24 h prior to TG treatment in the glucose condition and then 12 µM TG was added for 5 h. Cells were rinsed with cold PBS and harvested in 200 µl of NP40 lysis buffer (10 mM Tris, pH 7.5, 150 mM NaCl, 1 mM EDTA, 1 mM EGTA, 0.5% NP40, 1 mM PMSF, 10 µg/ml of protease inhibitors). Twenty microgram of each cell lysate was transferred into 96 well black wall plates, 200 µl of caspase assay solution (20 mM Hepes, pH 7.5, 10% glycerol, 2 mM DTT) containing 45 µM each caspase substrate [caspase-3 substrate (Ac-DEVD-AMC), caspase-8 substrate (Ac-IETD-AMC), or caspase-9 substrate (Ac-LEHD-AMC)] was added to each well, and the reaction mixture was incubated for 5 h at 37°C. Fluorescence was measured using Synergy HT plate reader (BioTek) with excitation at 360 nm and emission at 460 nm.

### Measurement of Mitochondrial Membrane Potential (ΔΨm)

ΔΨm was measured using 5,5′,6,6′-tetrachloro-1,1′,3,3-tetraethyl-benzimidazolyl-carbocyanine iodide (JC-1, Invitrogen) dye. Higher ΔΨm leads JC-1 to form aggregates that exhibit a red emission, while lower ΔΨm allows JC-1 to stay as a monomer that yields a green emission. Cells were incubated with the different metabolic media in the presence or absence of RSG with or without GW9662. Twenty four hours later, cells were treated with TG for the times indicated. After treatment, 5 µg/ml JC-1 was added. Plates were wrapped with aluminum foil and returned to the incubator. Thirty minutes later, cells were washed with PBS and fluorescence was measured on Synergy HT plate reader with the setup of green fluorescence (excitation at 485 nm and emission at 528 nm) and red fluorescence (excitation at 540 nm and emission at 590 nm). The ratio of red fluorescence to green fluorescence was calculated as ΔΨm and then JC-1 ratio from each treatment was normalized by JC-1 ratio from the control of each cell type.

### Intracellular Superoxide/ROS Measurement

Superoxide/ROS generation was measured using DHE, a cell-permeable reduced form of ethidium bromide which is not cell-permeable. DHE itself exhibits a blue fluorescence in cytoplasm. Oxidation especially by superoxide transforms DHE into oxidized DHE products which exhibit a red fluorescence. The red fluorescence of oxidized DHE products becomes much brighter after DNA intercalation. Twenty four hours after plating striatal cells, the media was replaced with the different metabolic media. Imaging experiments for DHE were performed after 24 h. Glucose medium was changed with G-HBSS consisting of HBSS (20 Hepes, pH 7.4, 137 NaCl, 5 KCl, 0.5 KH_2_PO_4_, 0.5 Na_2_HPO_4_, 10 NaHCO_3_, 0.01 glycine; in mM), 2 mM CaCl_2_, 0.6 mM MgCl_2_, and 10 mM glucose, and pyruvate medium was changed with Ox-HBSS consisting of HBSS, 2 mM CaCl_2_, 0.6 mM MgCl_2_, 5 mM galactose, 2 mM glutamine, and 1 mM pyruvate. 5 µM DHE was included in G-HBSS or Ox-HBSS. TG was added 10 min later. Plates were wrapped with aluminum foil and replaced in the incubator. 30 min later, oxidized DHE was imaged at 20× magnification on an Observer D1 microscope (Zeiss) coupled with a digital CCD camera (ORCA-ER, Hamamatsu Photonics). The red fluorescence from oxidized DHE was detected by using a 545/40 excitation filter and a 630/75 emission filter with the same exposure time among each experimental groups. DHE positive cells were counted using Image J software. Primary cortical neurons were transfected with 0.8 µg of plasmids as indicated using Lipofectamine 2000 on DIV 7. Five hours after transfection, the media was replaced with half conditioned NBM and half complete NBM. On DIV 13, neurons were rinsed with G-HBSS and maintained in G-HBSS containing 2.5 µM DHE. Plates were wrapped with aluminum foil and replaced in the incubator. One hour later, all GFP-positive neurons were examined for DHE staining and photographed with a 40× oil objective on the microscope system as described above. DHE positive neurons were counted when the red fluorescence occurred in the nucleus and its intensity was at least 50% higher than that in cytoplasm.

### Statistical Analysis

Data were expressed as mean ± SE (standard error) and analyzed using Student's *t* test except where noted. Statistical significance was considered when a *P* value was <0.05.

## Supporting Information

Figure S1 **Glycolysis may contribute to the increased sensitivity of STHdh^Q111^ cells to stressors.** Striatal cells were treated with either 10 µg/ml oligomycin or 10 µM rotenone to inhibit Oxphos in the glucose condition for 2 h prior to treatment with stressors. Cell viability was measured 8 h after treatment with H_2_O_2_ (*A*) or TG (*B*) using the resazurin assay. Oligomycin or rotenone treatment in the glucose condition led to significantly reduced viability of STHdh^Q111^ cells in response to H_2_O_2_ or TG compared to STHdh^Q7^ cells. *n* = 5. Data shown are mean ± SE. * *P*<0.05, ** *P*<0.01.
(TIF)

Figure S2 **STHdh^Q111^ cells in the low glucose (5 mM) condition still exhibit much greater cell death in response to stressors.** STHdh^Q7^ and STHdh^Q111^ cells were maintained in the low glucose condition for 24 h prior to treatment with stressors for 12 h. *A*, H_2_O_2_ treatment resulted in much greater cell death in STHdh^Q111^ than STHdh^Q7^ cells. *B*, TG treatment also resulted in significant cell death in STHdh^Q111^ cells while STHdh^Q7^ cells were resistant to given treatment. *n* = 2. Data shown are mean ± SD (standard deviation).
(TIF)

Figure S3 **Knockdown of PPARγ in STHdh^Q7^ cells does not increase sensitivity to stressors.** *A*, STHdh^Q7^ cells stably expressing shRNA for PPARγ were generated and show significantly reduced PPRE activity compared to naïve STHdh^Q7^ cells. n = 3–4. *B*, Reduced PPARγ activity in STHdh^Q7^ cells does not aggravate cell death in response to H_2_O_2_ or TG in the glucose condition. *n* = 3–6. Data shown are mean ± SE.
(TIF)

Figure S4 **STHdh^Q111^ cells show a significant reduction in the expression of Bcl-2 and cyclophilin D compared to STHdh^Q7^ cells.** Cells were maintained for 24 h in the glucose condition and harvested for either western blot or real-time PCR as described in Experimental Procedures. *A*, Immunoblot results show significantly reduced protein expression of Bcl-2 and cyclophilin D (CypD) in STHdh^Q111^ cells compared to STHdh^Q7^ cells. *n* = 4. *B*, The mRNA level of CypD is significantly reduced in STHdh^Q111^ cells. *n* = 4. *C*, STHdh^Q7^ and STHdh^Q111^ cells were incubated in the presence or absence of 20 µM RSG for 24 h. RSG treatment does not change the protein expression of PPARγ, Bcl-2, CypD, or VADC. Data shown are mean ± SE. * *P*<0.05, *** *P*<0.001 vs. STHdh^Q7^.
(TIF)

Figure S5 **TG-induced cell death may not involve ER stress or UPR response in the given condition.** STHdh^Q7^ and STHdh^Q111^ cells were treated with and without 20 µM RSG for 24 h in the glucose condition, and then 12 µM TG was treated for 3 h or 5 h. Cells were harvested and prepared for western blot analysis. TG treatment does not induce activation of caspase 12 or increase expression of BiP/GRP78 in both cell types in the given period of time. Interestingly, STHdh^Q111^ cells exhibit higher level of pro- and active caspase 12 compared to STHdh^Q7^ cells. RSG treatment does not have impact on either caspase 12 activation or BiP/GRP78 induction.
(TIF)

Figure S6 **The pyruvate condition results in a slight change in gene expression profile as compared to the glucose condition.** STHdh^Q7^ and STHdh^Q111^ cells were maintained in the pyruvate condition for 24 h. Real-time PCR was performed as described in materials and methods. *n* = 4. The relative mRNA levels of STHdh^Q111^ cells to the corresponding each gene of STHdh^Q7^ cells were plotted. mRNA levels of PPARγ and UCP4 are decreased and mRNA level of UCP2 is highly increased in STHdh^Q111^ cells as shown in the glucose condition. Similarly, mRNA levels of PGC-1a and SIRT1 are not different between two cell types as shown in the glucose condition. However, there is no difference in mRNA levels of CytC, SOD1, SOD2, and SERCA2 between two cell types, which were shown to be decreased in STHdh^Q111^ cells. Data shown are mean ± SE. * *P*<0.
(TIF)
