# Supplementary figures and images for: Metabolic State Determines Sensitivity to Cellular Stress in Huntington Disease: Normalization by Activation of PPARγ
Source: PLoS One. 2012 Jan 20;7(1):e30406. doi: 10.1371/journal.pone.0030406 (PMC3262812; doi:10.1371/journal.pone.0030406)

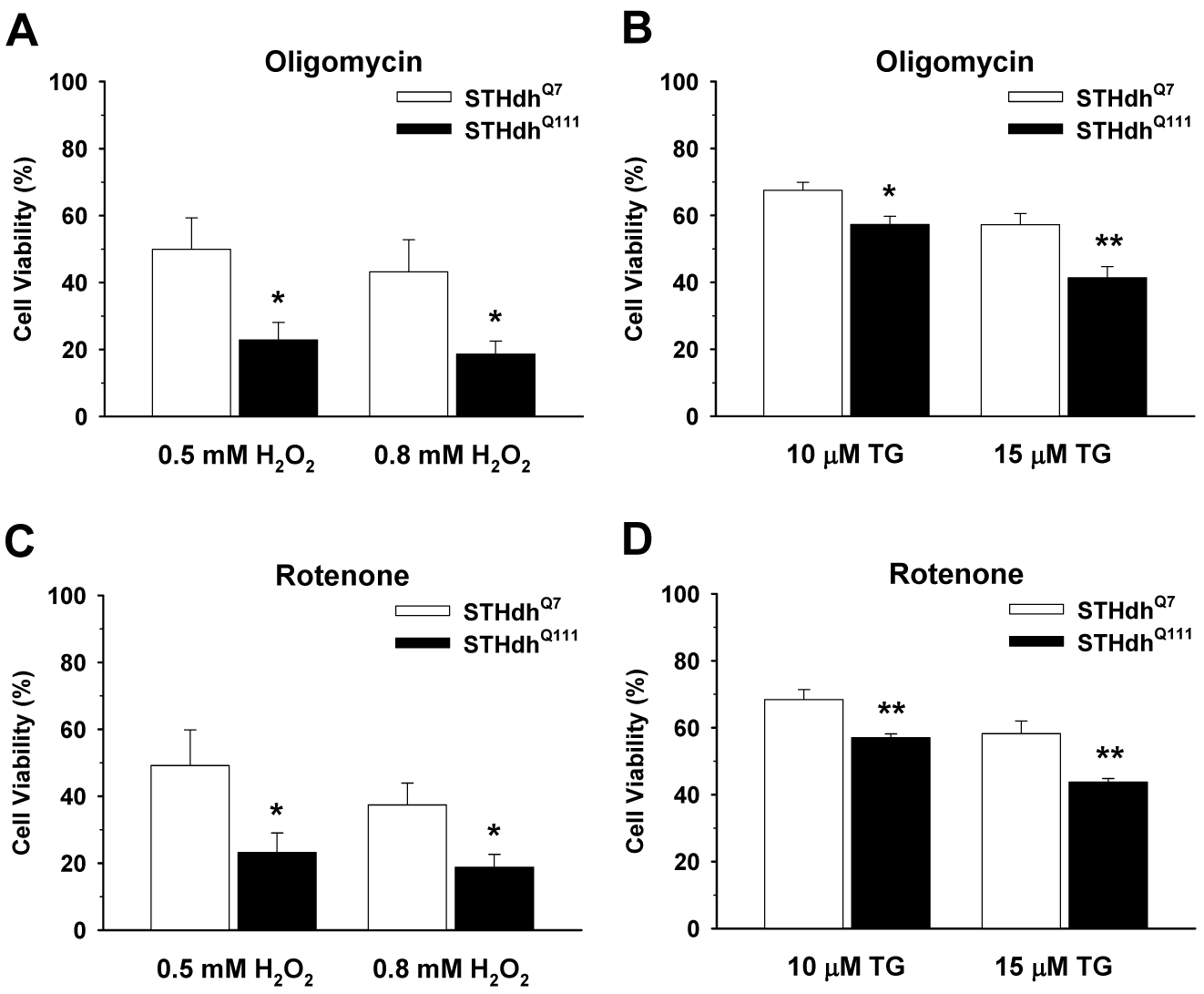

Supplement: Figure S1 — Glycolysis may contribute to the increased sensitivity of STHdhQ111 cells to stressors. Striatal cells were treated with either 10 µg/ml oligomycin or 10 µM rotenone to inhibit Oxphos in the glucose condition for 2 h prior to treatment with stressors. Cell viability was measured 8 h after treatment with H2O2 (A) or TG (B) using the resazurin assay. Oligomycin or rotenone treatment in the glucose condition led to significantly reduced viability of STHdhQ111 cells in response to H2O2 or TG compared to STHdhQ7 cells. n = 5. Data shown are mean ± SE. * P<0.05, ** P<0.01. (TIF) [file pone.0030406.s001.tif]

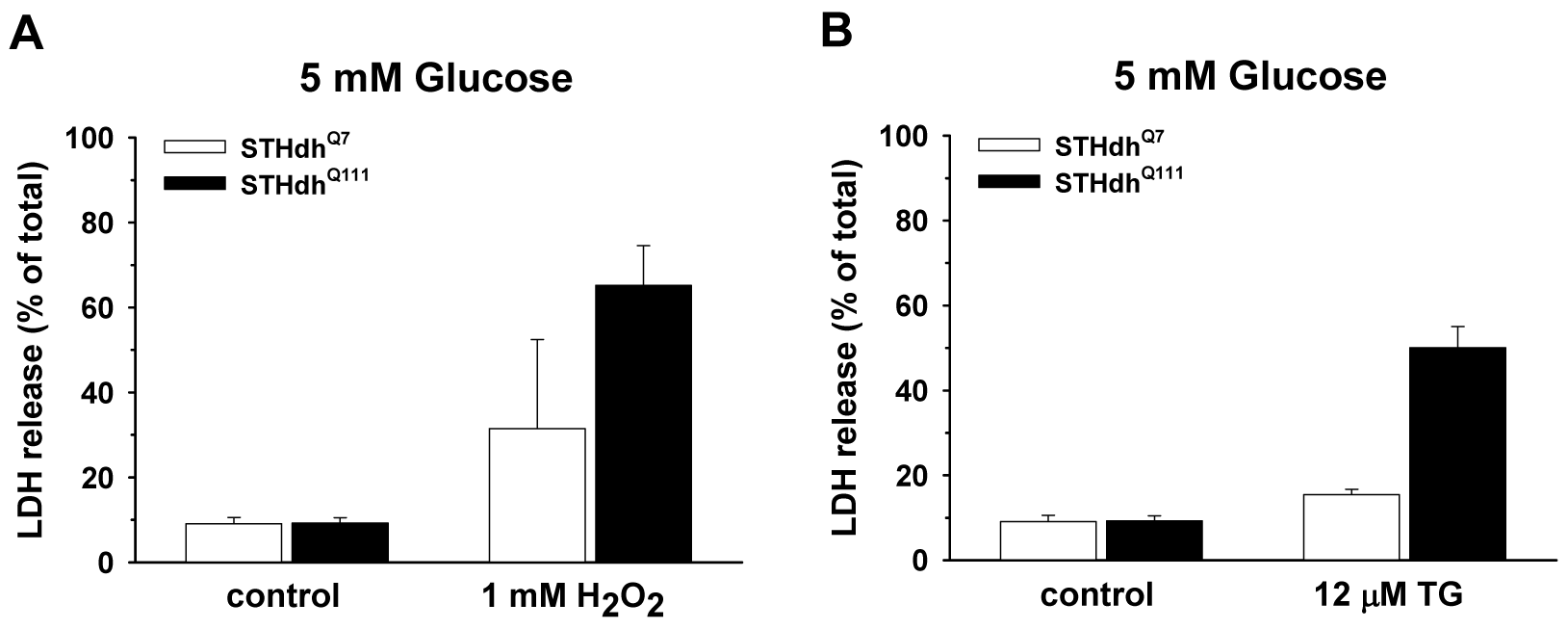

Supplement: Figure S2 — STHdhQ111 cells in the low glucose (5 mM) condition still exhibit much greater cell death in response to stressors. STHdhQ7 and STHdhQ111 cells were maintained in the low glucose condition for 24 h prior to treatment with stressors for 12 h. A, H2O2 treatment resulted in much greater cell death in STHdhQ111 than STHdhQ7 cells. B, TG treatment also resulted in significant cell death in STHdhQ111 cells while STHdhQ7 cells were resistant to given treatment. n = 2. Data shown are mean ± SD (standard deviation). (TIF) [file pone.0030406.s002.tif]

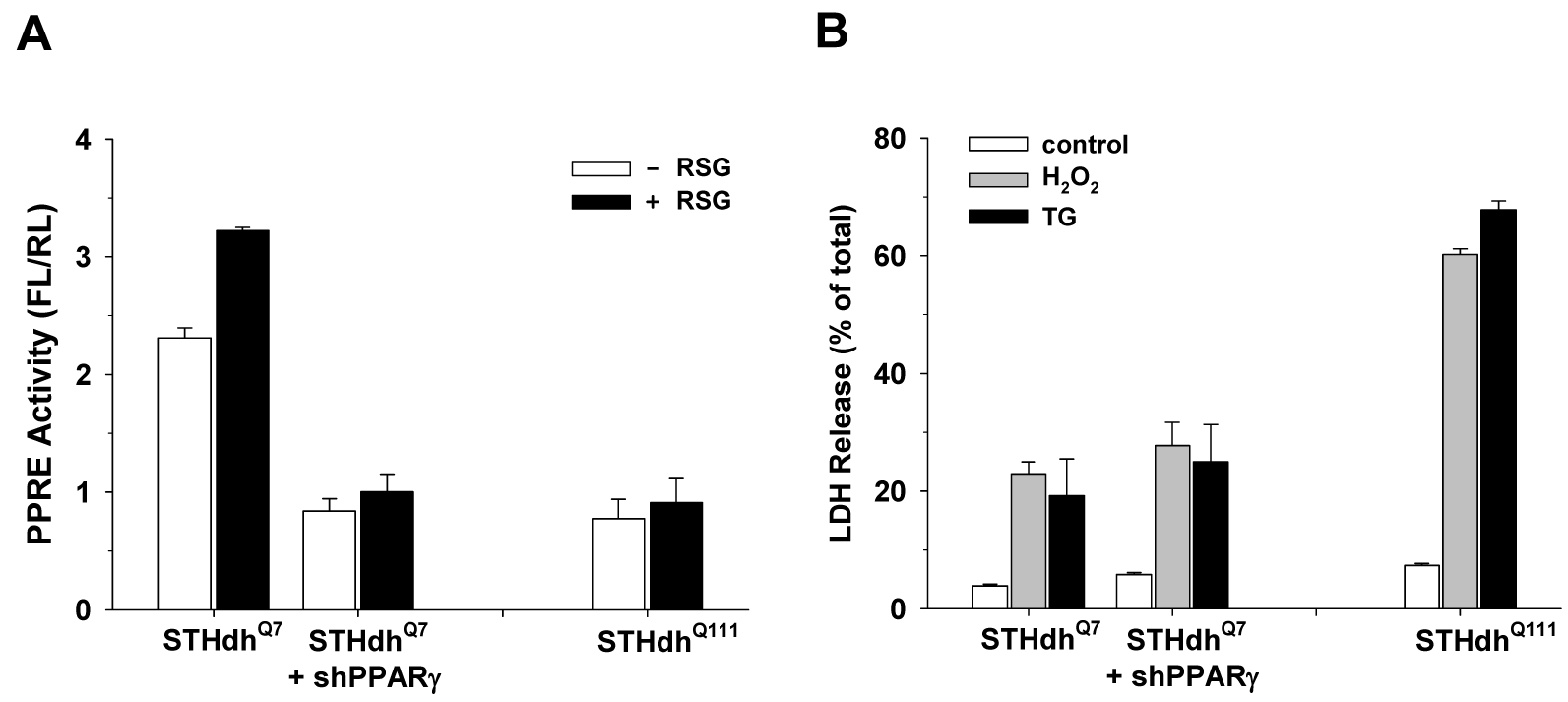

Supplement: Figure S3 — Knockdown of PPARγ in STHdhQ7 cells does not increase sensitivity to stressors. A, STHdhQ7 cells stably expressing shRNA for PPARγ were generated and show significantly reduced PPRE activity compared to naïve STHdhQ7 cells. n = 3–4. B, Reduced PPARγ activity in STHdhQ7 cells does not aggravate cell death in response to H2O2 or TG in the glucose condition. n = 3–6. Data shown are mean ± SE. (TIF) [file pone.0030406.s003.tif]

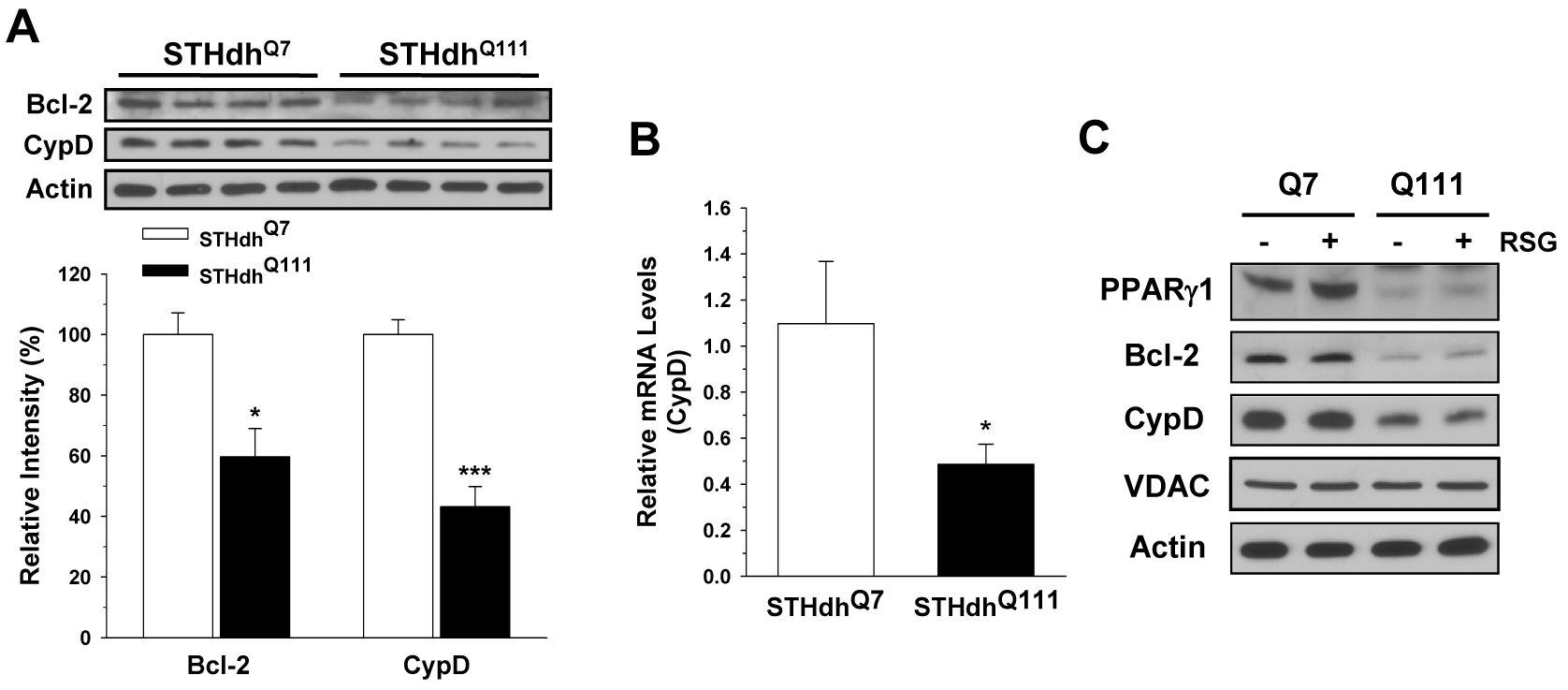

Supplement: Figure S4 — STHdhQ111 cells show a significant reduction in the expression of Bcl-2 and cyclophilin D compared to STHdhQ7 cells. Cells were maintained for 24 h in the glucose condition and harvested for either western blot or real-time PCR as described in Experimental Procedures. A, Immunoblot results show significantly reduced protein expression of Bcl-2 and cyclophilin D (CypD) in STHdhQ111 cells compared to STHdhQ7 cells. n = 4. B, The mRNA level of CypD is significantly reduced in STHdhQ111 cells. n = 4. C, STHdhQ7 and STHdhQ111 cells were incubated in the presence or absence of 20 µM RSG for 24 h. RSG treatment does not change the protein expression of PPARγ, Bcl-2, CypD, or VADC. Data shown are mean ± SE. * P<0.05, *** P<0.001 vs. STHdhQ7. (TIF) [file pone.0030406.s004.tif]

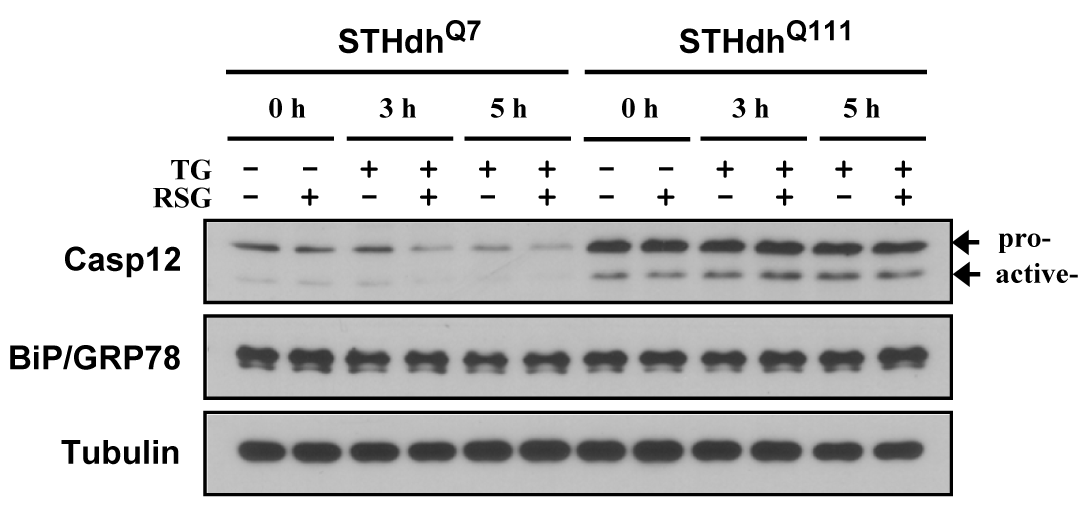

Supplement: Figure S5 — TG-induced cell death may not involve ER stress or UPR response in the given condition. STHdhQ7 and STHdhQ111 cells were treated with and without 20 µM RSG for 24 h in the glucose condition, and then 12 µM TG was treated for 3 h or 5 h. Cells were harvested and prepared for western blot analysis. TG treatment does not induce activation of caspase 12 or increase expression of BiP/GRP78 in both cell types in the given period of time. Interestingly, STHdhQ111 cells exhibit higher level of pro- and active caspase 12 compared to STHdhQ7 cells. RSG treatment does not have impact on either caspase 12 activation or BiP/GRP78 induction. (TIF) [file pone.0030406.s005.tif]

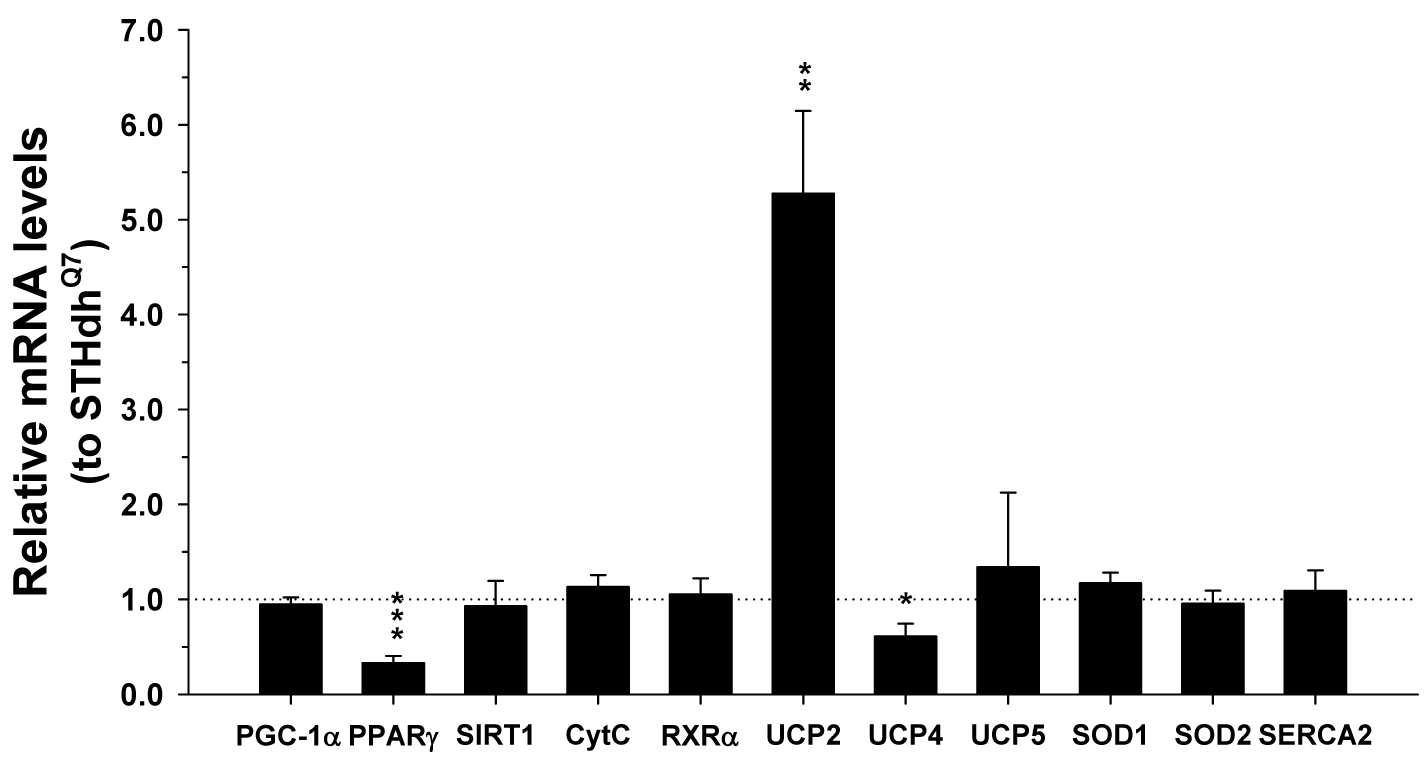

Supplement: Figure S6 — The pyruvate condition results in a slight change in gene expression profile as compared to the glucose condition. STHdhQ7 and STHdhQ111 cells were maintained in the pyruvate condition for 24 h. Real-time PCR was performed as described in materials and methods. n = 4. The relative mRNA levels of STHdhQ111 cells to the corresponding each gene of STHdhQ7 cells were plotted. mRNA levels of PPARγ and UCP4 are decreased and mRNA level of UCP2 is highly increased in STHdhQ111 cells as shown in the glucose condition. Similarly, mRNA levels of PGC-1a and SIRT1 are not different between two cell types as shown in the glucose condition. However, there is no difference in mRNA levels of CytC, SOD1, SOD2, and SERCA2 between two cell types, which were shown to be decreased in STHdhQ111 cells. Data shown are mean ± SE. * P<0. (TIF) [file pone.0030406.s006.tif]
